# Disparities in hepatitis B virus healthcare service access among marginalised poor populations: a mixed-method systematic review

**DOI:** 10.1186/s40249-024-01225-0

**Published:** 2024-08-09

**Authors:** Caixia Li, Dejina Thapa, Qian Mi, Yuanxiu Gao, Xia Fu

**Affiliations:** 1 The Department of Nursing, The Eighth Affiliated Hospital, Sun Yat-sen University, Shenzhen, Guangdong Province China; 2 Nepal Health Research Council, Kathmandu, Nepal; 3 The School of Nursing, Guangdong Pharmaceutical University, Guangzhou, China

**Keywords:** Hepatitis B virus, Health disparity, Marginalised poor, Poverty, Systematic review

## Abstract

**Background:**

Marginalised poor populations, characterised by poverty and social exclusion, suffer disproportionately from hepatitis B virus (HBV) infections and encounter substantial disparities in access to healthcare. This has further exacerbated the global HBV burden and precluded progress towards HBV elimination. This mixed-method systematic review aimed to synthesise their utilisation and influencing factors in HBV healthcare services, including screening, vaccination, treatment, and linkage-to-care.

**Methods:**

Eleven databases were searched from their inception to May 4, 2023. Quantitative and qualitative studies examining the factors influencing HBV healthcare access among marginalised poor populations were included. A meta-analysis was conducted to synthesise the pooled rates of HBV healthcare utilisation. The factors influencing utilisation were integrated and visualised using a health disparity research framework.

**Results:**

Twenty-one studies were included involving 13,171 marginalised poor individuals: sex workers, rural migrant workers, irregular immigrants, homeless adults, and underprivileged individuals. Their utilisation of HBV healthcare ranged from 1.5% to 27.5%. Meta-analysis showed that the pooled rate of at least one dose of the HBV vaccine barely reached 37% (95% confidence interval: 0.26‒0.49). Fifty-one influencing factors were identified, with sociocultural factors (*n* = 19) being the most frequently reported, followed by behavioural (*n* = 14) and healthcare system factors (*n* = 11). Socio-cultural barriers included immigration status, prison history, illegal work, and HBV discrimination. Behavioural domain factors, including previous testing for sexually transmitted diseases, residential drug treatment, and problem-solving coping, facilitated HBV healthcare access, whereas hostility coping exerted negative influences. Healthcare system facilitators comprised HBV health literacy, beliefs, and physician recommendations, whereas barriers included service inaccessibility and insurance inadequacies. The biological and physical/built environments were the least studied domains, highlighting that geographical mobility, shelter capacity, and access to humanitarian health centres affect HBV healthcare for marginalised poor populations.

**Conclusions:**

Marginalised poor populations encounter substantial disparities in accessing HBV healthcare, highlighting the need for a synergistic management approach, including deploying health education initiatives to debunk HBV misperceptions, developing integrated HBV management systems for continuous tracking, conducting tailored community outreach programmes, and establishing a human rights-based policy framework to guarantee the unfettered access of marginalised poor populations to essential HBV services.

**Supplementary Information:**

The online version contains supplementary material available at 10.1186/s40249-024-01225-0.

## Background

The hepatitis B virus (HBV) is a hepatotropic DNA virus that affects nearly one-third of the global population [1]. HBV caused approximately 254 million chronic infections in 2022 [1], which was more than HIV, tuberculosis, or malaria combined [2, 3]. The number of HBV-related deaths from HBV are projected to increase by 35% from 820,000 in 2019 to 1,109,500 by 2030 [4]. HBV persists as a substantial public health concern across global regions (e.g. Asia-Pacific and sub-Saharan Africa). The World Health Assembly has endorsed the goal of eliminating HBV, defined as a 65% reduction in mortality and a 90% decline in the incidence of hepatitis B between 2015 and 2030 [5]. To achieve this, at least 90% of patients with hepatitis B must be diagnosed, and 80% of eligible patients must be treated [5]. However, HBV elimination activities, from prevention to testing and treatment, receive insufficient attention and investment, and were only funded by 37% of countries by 2017 [6].

Health equity alongside HBV elimination is even more been neglected. HBV is unevenly distributed across societal strata, with a disproportionately higher prevalence in marginalised poor populations who experience poverty and social exclusion from economic, social, political, and cultural dimensions [7]. These groups include, but are not limited to, homeless people, disabled individuals, sanitation workers, commercial sex workers, rural-urban migrant workers, incarcerated individuals, and irregular migrants such as refugees and asylum seekers [8]. A recent meta-analysis reported an estimated HBV prevalence of 15% among sanitation workers from Asian, African, and South American regions [9], compared with approximately 4.1% in the general population globally [3]. A prevalence rate of 30.9% for HBV exposure and incidences 7 to 10 times higher for HBV prevalence have been estimated among homeless individuals from the United States [10]. The prevalence rates among sex workers were reported to be 9.2% [11] and as high as 13.6% to 60.8% in refugees from low- and middle-income countries, including Ethiopia, Thailand, and Pakistan [12]. Unstable living conditions, poor living standards, limited access to healthcare, and exposure during work (e.g. biological exposure during waste picking) create a permissive environment for HBV transmission among the marginalised poor [8, 9].

HBV healthcare services, ranging from vaccination to screening, treatment, and linkage-to-care, are strikingly less accessible to marginalised poor populations. Only 16.7% to 38.7% of the marginalised population, including female sex workers [13], homeless individuals, and those incarcerated [14], exhibited a serological profile of HBV vaccination. More importantly, the asymptomatic nature of chronic HBV infection necessitates a reliance on screening to identify cases. However, HBV screening has been poorly utilised, with only 10.5% of those infected with HBV worldwide aware of their status, and a mere 2.2% receiving treatment in 2019 [1]. These figures are suspected to be even lower among the marginalised poor due to insufficient data capture and multiple access barriers to services, including low health literacy, competing life priorities (food, clothing, and shelter), and difficulties in accessing healthcare facilities (e.g. lack of insurance, long-distance travel, and fear of judgment by health professionals) [15]. Reports suggest that only 1.5% of underprivileged individuals living in shelters had completed HBV screening, resulting in substantial delays in HBV diagnosis and treatment [16]. As a result, marginalised poor populations suffer more complications and mortality from HBV infection compared to the general public [8].

Despite this, factors influencing HBV healthcare access among marginalised poor populations are scarcely represented and synthesised in previous research, precluding an in-depth understanding of health needs and further allocation of health resources towards HBV elimination among the population. Thus, this review was conducted to synthesise the evidence on HBV healthcare service utilisation and its influencing factors among marginalised poor populations. It was guided by the National Institute on Minority Health and Health Disparity (NIMHD) research framework, which employs an integrative approach to represent multifaceted levels (individual, interpersonal, community, and societal) and domains (biological, behavioural, physical/built environment, socio-cultural environment, and healthcare system) that collectively explain health disparities [17]. The multitude of factors will be synthesised and visualised in the NIMHD framework to inform tailored interventions, policy-making, and resource allocation towards the global HBV elimination goal.

## Methods

A systematic review and meta-analysis were conducted. The protocol was registered with the International Prospective Register of Systematic Reviews (CRD42022381183).

### Literature search

Following the PICOs framework, approximately 133 search terms (Supplementary material 1) were developed pertaining to population and outcomes of this review, including “marginalized poor”, “hepatitis B”, “screening”, “vaccination”, “linkage-to-care”, and “influencing factors”. Relevant synonyms (e.g. hard to reach) and medical subject-heading (MeSH) terms were identified by referencing previous literature on marginalised poor populations [18] and by conducting an initial search in MEDLINE via OvidSP.

Search terms were retrieved in the fields of “title”, “keywords”, and “abstract”. Truncations (*) and adjacency searchers (adj) were used to enhance search efficiency. The following 11 databases were searched from their inception to May 4, 2023: Embase, MEDLINE via Ovid, Ovid Emcare, Ovid Nursing Database, British Nursing Index, Ovid APA PsycInfo, Cochrane Library, CINAHL, ProQuest Health & Medicine Collection, Scopus, and China National Knowledge Internet. There were no restrictions on language or publication data. A medical librarian refined the search strategy. Detailed search records for each database are shown in Supplementary material 1.

### Study screening

After removing duplicates using Covidence (Veritas Health Innovation, Melbourne, Australia), four researchers independently screened the titles and abstracts of the retrieved articles against the eligibility criteria. The full texts of potentially eligible articles were retrieved and scrutinised by pairs of researchers. Disagreements were resolved through discussion.

### Inclusion and exclusion criteria

Studies were eligible if they reported factors influencing HBV healthcare access among marginalised poor populations. The ‘Participant-Intervention-Comparator-Outcomes-Study design’ (PICOs) framework [19] was followed to formulate the following eligibility criteria:

#### Population

The marginalised poor, who experience poverty and social exclusion across economic, social, political, and cultural dimensions, were eligible [7]. Focusing on the specific attributes of marginalisation—low-skill levels, low-socioeconomic status, and disability—this review included the following groups of marginalised poor individuals aged ≥ 18 years: (1) homeless adults; (2) migrant workers, including migrant domestic workers, migrant farmworkers, and migrant construction workers; (3) individuals with low socioeconomic status, such as farmers, construction workers, sanitation workers, the unemployed, and those living in poverty; and (4) individuals with disabilities. However, other marginalised populations, including men who have sex with men, were outside the scope of this review, and only included migrants with low socioeconomic status. Studies that included both the marginalised poor and the general population were eligible only if a subgroup analysis of the former was performed.

#### Outcomes

Studies should report factors influencing poor marginalised populations engagement in HBV vaccination, screening, treatment, and linkage-to-care, which refers to the process of referring patients with hepatitis B to medical care, ensuring that they receive directed care and treatment and are monitored regularly [20].

#### Study design

Quantitative, qualitative, and mixed-methods studies were eligible if they met the aforementioned criteria. Reviews, conference abstracts, editorials, guidelines, and letters were excluded.

### Data extraction

A standard data extraction form was used to extract study data, focusing on factors influencing HBV healthcare access among marginalised poor populations. Odds ratios (*OR*s), 95% confidence intervals (*CI*s), and *P* values were extracted whenever possible to identify significant influencing factors. Insignificant factors were also extracted for study comparison. Qualitative data were summarised narratively. The extracted data were checked and validated by other researchers.

### Critical appraisal of methodological quality

The Mixed Methods Appraisal Tool Version 2018 was used to assess the methodological quality of the included studies [21]. Two screening items assessing the clarity of the research questions and their coherence with the collected data were applied to all included studies. Five further questions were appraised based on study types. For quantitative descriptive studies, these five questions assessed the sampling strategy, representativeness of study samples, nonresponse bias, measurements, and statistical methods. For qualitative studies, the appropriateness of the qualitative approach, qualitative data collection methods, data analysis, interpretation, and coherence between them were assessed. Each item was rated as Yes, No, or Cannot Tell. One point was given for the Yes rating and zero for the other ratings. Total scores were converted into percentage scores. Three authors independently conducted quality appraisals, and any discrepancies in ratings were resolved through discussion.

### Data synthesis

Factors influencing HBV healthcare access among marginalised poor populations were integrated based on the NIMHD framework into five domains: biological, physical/built environment, behavioural, socio-cultural environment, and healthcare system. Within each domain, the influence from the individual to interpersonal, community, and societal levels was categorised and analysed whenever applicable. The STATA 18.0 (StataCorp LLC, College Station, Texas, USA) was used to conduct meta-analyses to synthesise the rate of HBV healthcare utilisation and generate the combined effects of the influencing factors. Pooled rates, *OR*, and 95% *CI* were calculated, with the significance level set at *P* < 0.05. Heterogeneity was evaluated using Cochrane’s Q test, with *P* < 0.1 indicating significant heterogeneity [22]. When heterogeneity was statistically significant, a random-effects model using the DerSimonian-Laird method was used; otherwise, a fixed-effects model was used.

## Results

### Search results

**Fig. 1:**
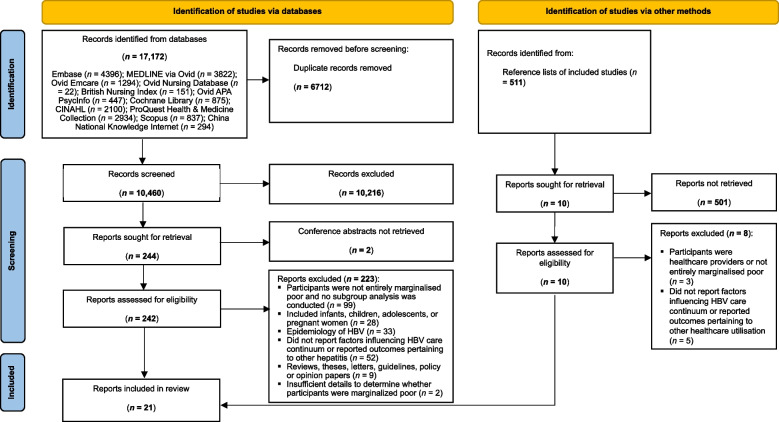
The Preferred Reporting Items for Systematic Reviews and Meta-analyses (PRISMA) diagram

The database search identified 17,172 articles (Fig. 1). A total of 6712 duplicates were removed, and 10,216 articles were excluded after title and abstract screening. Among the 242 full-text articles retrieved, 223 were excluded mainly because they did not entirely focus on marginalised poor populations or report factors influencing HBV healthcare utilisation. Two additional studies were included after screening the references. Ultimately, 21 studies were included in the analysis [16, 23–42].

### Study characteristics

Nineteen quantitative descriptive studies and two qualitative studies published between 2007 and 2021 (Table 1) were included. More than half of these studies were conducted in the United States of America (*n* = 7), Brazil (*n* = 3), China (*n* = 2), and France (*n* = 2). The marginalised poor populations in these countries included: (1) sex workers (*n* = 6); (2) rural migrant workers (*n* = 2); (3) irregular, low income, and underserved immigrants (*n* = 5); (4) homeless adults, parolees, and prisoners (*n* = 4); and (5) underprivileged and low socio-economic status individuals (*n* = 4), such as collectors of recyclable waste and roadside barbers.

**Table 1:** Characteristics of included studies (*n* = 21)

**Author, year, and country**	**Study design, setting and context**	**Marginalised poor populations**	**Utilisation of HBV healthcare service**	**Factors influencing HBV healthcare service access among marginalised poor populations**
Baars et al. 2009 [23], Netherlands	A cross-sectional study conducted at prostitution locations, including brothels. A nationwide HBV vaccination program was commenced in 2002 for commercial female sex workers.	Commercial female sex workers (*n* = 259)	Received at least one dose of HBV vaccination: 63.4% (163/257)	**Factors influencing HBV vaccination uptake** (1) **Information from professionals.** Receiving information about HBV vaccination programme was a strong predictor of HBV vaccination uptake among female sex workers (*OR* = 4.27, 95% *CI*:1.84‒9.92). (2) **Duration of working**. Duration of working in current prostitution was marginally associated with HBV vaccine uptake of at least one dose among female sex workers (*OR* = 1.01, 95% *CI*: 1.00‒1.03). (3) **Region**. Female sex workers residing in South Limburg had higher odds of undertaking the HBV vaccine for at least one dose compared to those residing in Rotterdam (*OR* = 4.09, 95% *CI*: 1.23‒13.59). (4)**Factors examined without showing a significant association**. Age, religious, frequency of client contacts, and window prostitution. **Reasons for not undergoing HBV vaccination** (1) **Lack of time or geographic mobility**. Lack of time and moving to another workplace were important reasons for female sex workers not obtaining HBV vaccination. (2) **Lack of knowledge and awareness**. Lack of knowledge regarding hepatitis B, vaccine, vaccination procedure, and unawareness of free three-dose hepatitis B vaccination also precluded female sex workers from receiving HBV vaccination. (3) **Others.** Believed that they would not become infected with HBV, were afraid of needles, and forgetting the appointment also discouraged female sex workers from undertaking HBV vaccination.
Carneiro et al. 2014 [24], Brazil	A longitudinal study conducted at prostitution settings. HBV vaccine was offered free-of-charge to female sex workers.	Female sex workers (*n* = 721)	(1) Previous HBV vaccination rate: 27.6% (199/721) (1) Three-dose HBV vaccine completion rate after outreach programme offering vaccine doses: 37.5% (146/389)	**Factors influencing completion of three-dose HBV vaccine** (4) **Formal educational year.** Female sex workers who had less than nine years of formal education had higher three-dose HBV vaccine completion rates (*adjPR* = 1.6, 95% *CI*: 1.1‒2.5) than those had nine or more years of formal education. (5) **Working place.** Female sex workers who worked on the street (*adjPR* = 1.9, 95% *CI*: 1.0‒3.7) and in massage parlors or brothels (*adjPR* = 2.4, 95% *CI*: 1.3‒4.6) had higher three-dose HBV vaccine completion rates than those worked at erotic show houses. (6) **Years of prostitution.** Female sex workers who had 1 to 5 years (*adjPR* = 2.1, 95% *CI*: 1.2‒3.9) or more than five years of prostitution (*adjPR* = 1.9, 95% *CI*: 1.0‒3.1) had higher HBV vaccine completion rates than those had less than one year of prostitution.
Freeland et al. 2021 [25], USA	A qualitative study conducted in the Vietnamese nail salon community, where many nail salon workers were from high-risk and underserved communities.	Vietnamese nail salon workers from underserved communities (*n* = 19)	N.A.	**Barriers and facilitators to HBV prevention, testing, treatment, and care** (1) **Lack of HBV knowledge**. Several Vietnamese nail salon workers had not heard about HBV and many of them had misperceptions about its transmission, symptoms, and prevention. For example, many did not know HBV vaccines are available to prevent HBV infection. (2) **Service access barriers**. Afraid of detecting diseases was a common barrier to healthcare service access. In addition, nail technicians have to work seven days a week. It is not convenient and time consuming to see a doctor. Further, without symptoms, healthcare cost, and lack of insurance also preclude them from receiving HBV care. (3) **Not being recommended by doctors**. Lack of doctors’ recommendation precluded them from undertaking HBV testing. Some participants misperceived that a blood test at their primary visit usually includes HBV testing and believed that they do not have HBV if doctors said that their blood tests were normal. (4) **Nail technician training and licensing process did not convey information of HBV**. Formal nail training usually contains fungus infections or bacteria and did not contain information about HBV transmission or prevention. They might also be misinformed that HBV can be prevented by wearing masks. (5) **Discrimination and stigma**. Owing to the knowledge gaps in HBV transmission and fear of infection, Vietnamese nail salon workers believed that patients with HBV were treated differently. People would avoid close contacts with patients with HBV. Family members would also be separated to avoid transmitting the virus to other family members. This appears to be a barrier to HBV prevention among the community. (6) **Public health campaigns**. Postings, leaflets, or brochures regarding HBV in Vietnamese communities, including supermarkets, nail salons, newspapers, churches, and templates, would be impactful to increase awareness and link them to community resources regarding HBV vaccination.
Jung et al. 2010 [26], USA	A retrospective study at a publicly-funded healthcare network.	Low-income immigrants (*n* = 1,231)	(1) HBV screening rate (HBV e antigen or HBV DNA testing): 27.5% (339/1231) (2) HBV treatment: 15.9% (110/691)	**Factors influencing HBV testing** (1) **Sex**. Male sex was negatively associated with HBV testing compared to female sex (*aOR* = 0.73, 95% *CI*: 0.55‒0.97, *P* = 0.028). (2) **Gastroenterology clinic visits**. Having more visits to gastroenterology clinics enhanced the odds of HBV testing (*aOR* = 2.6, 95% *CI*: 2.0‒3.3, *P* < 0.001). (3)**Healthcare contacts**. A less recent healthcare contact was positively associated with being tested for HBV (*aOR* = 4.4, 95% *CI*: 3.1‒6.3, *P* < 0.001). (4) **Factors examined without showing a significant association.** Age at time of HBV detection. **Factors influencing HBV treatment** (1) **HIV status**. HIV positive status increased the odds of HBV treatment (*aOR* = 26.7, 95% *CI*: 13‒53, *P* < 0.001). (2) **Liver biopsy**. Having had a liver biopsy was associated with HBV treatment (*aOR* = 5.4, 95% *CI*: 2.1‒14, *P* < 0.001). (3) **HBV testing**. Determination of HBeAg or HBV–DNA was positively associated with HBV treatment (*aOR* = 2.2, 95% *CI*: 1.1‒4.5, *P* = 0.033). (4) **Duration of known HBV infection status**. A longer duration of known HBV status was associated with a higher odds of HBV treatment (*aOR* = 1.1, 95% *CI*:1.03‒1.2, *P* = 0.013). (5) **Gastroenterology clinic visits**. Having a gastroenterology clinic visit enhanced the odds of HBV treatment (*aOR* = 1.4, 95% *CI*: 1.2‒1.6,* P* < 0.001). (6) **Most recent healthcare contact**. A less recent healthcare contact was negatively associated with HBV treatment (*aOR* = 0.39, 95% *CI*: 0.17‒0.89, *P* = 0.026). (7) **Factors examined without showing a significant association**. Sex, ethnicity, age at time of HBV detected, and lowest serum albumin level.
Liu et al. 2016 [27], China	A cross-sectional survey (study setting was not specified).	Rural migrant workers (*n* = 1,684)	HBV vaccination rate: 27.7% (467/1684) at least one dose	**Factors influencing HBV vaccination** (1) **Perceived vulnerability**. Perceived vulnerability increased the odds of rural migrant workers undertaking at least one dose of HBV vaccination (*OR* = 1.395, 95% *CI*: 1.181‒1.647, *P* < 0.001). (2) **Perceived efficacy**. A higher perceived efficacy of HBV vaccination increased the likelihood of rural migrant workers receiving at least one vaccination dose (*OR* = 1.211, 95% *CI*: 1.040‒1.410, *P* = 0.014). (3) **Age**. Rural migrant workers who aged 26-35 years (*OR* = 0.306, 95% *CI*: 0.233‒0.401, *P* < 0.001), 36-45 (*OR* = 0.139, 95% *CI*: 0.094‒0.206, *P* < 0.001) years, or ≥ 46 years (*OR* = 0.061, 95% *CI*: 0.034‒0.110, *P* < 0.001) had lower odds of undertaking at least one vaccination dose than those aged 16-25 years. (4) **Educational level**. Rural migrant workers who had a high education level had higher odds of undergoing at least one vaccination dose compared to those with a primary education level (*OR* = 1.483, 95% *CI*: 1.122‒1.960, *P* = 0.006). (5) **Distance to health services**. Rural migrant workers who had near (*OR* = 3.530, 95% *CI*: 1.755‒7.103, *P* < 0.001) or middle (*OR* = 3.547, 95% *CI*: 1.725‒7.293, *P* = 0.001) distance to health services were more likely to be vaccinated against HBV with at least one dose compared to those who had a long distance to health services. (6) **Factors examined without showing a significant association**. Perceived disease severity, self-efficacy, sex, income level, medical insurance, and self-related health status.
Magalhães et a. 2017 [28], Brazil	A cross-sectional study conducted in the city of Teresina. HBV vaccine was offered free-of-charge to female sex workers.	Female sex workers (*n* = 402)	(1) Self-reported HBV vaccination: 29.4% (118/402) (2) With free HBV vaccination, the first, second, and third doses were 90.8% (258/284), 60.8% (157/258), and 26.3% (68/258), respectively	**Factors influencing non-completion of three-dose HBV vaccination** (1) **Working place**. Female sex workers who worked in clubs had higher odds of not completing the three-dose HBV vaccine than those worked in bars, streets, or squares (*aOR* = 23.21, 95% *CI*: 3.08‒175.39, *P* = 0.002). (2) **Illicit drug utilisation**. Female sex workers who used illicit drugs had a 2.19 (95% *CI*: 1.11‒4.31, *P* = 0.024) times more likely to not complete the HBV vaccination schedule than those who had not used illicit drugs. (3) **Factors examined without showing a significant correlation**: age, piercing, having another professional occupation. **Reasons for non-completion of three-dose HBV vaccination** (1) **Geographic mobility**. Geographic mobility (*n* = 155) was expressed by female sex workers as a main reason for non-completion of the three-dose HBV vaccination. (2) **Other reasons**. Other reasons included having no free time on the HBV vaccine scheduled dates (*n* = 4), being detained in prisons (*n* = 4), being hospitalised (*n* = 3), or quitting prostitution (*n* = 11).
Mayanja et al. 2019 [29], Uganda	A prospective study conducted in a clinic. Free HBV vaccine was offered in the study.	Female sex workers (*n* = 345)	With free HBV vaccination, the three-dose HBV vaccine completion rate was 82.4% (239/290)	**Factors influencing non-completion of HBV vaccine series** (1) **Age**. Female sex workers aged 18-34 years were more likely to miss at least one HBV vaccine dose than those aged > 35 years (*IRR* = 13.10, 95% *CI*: 1.89‒90.92). (2) **Genital ulcer disease**. Female sex workers who reported to have genital ulcer disease had higher risks of missing at least HBV vaccine dose than those who did not (*IRR* = 3.02, 95% *CI*: 1.71‒5.33). (3) **Consistent condom utilisation**. Female sex workers who consistently used condoms were more likely to miss at least one HBV vaccine dose than those who did not (*IRR* = 2.57, 95% *CI*: 1.10‒6.07). (4) **Factors examined without showing a significant correlation.** Educational level, marital status, alcohol and illicit drug utilisation, being drunk before sex, reported vaginal discharge syndrome, number of sexual partners, new sex partners in the past three months, and paying for sex in the past 3 months.
Mazzitelli et al. 2021 [30], Italy	A retrospective study conducted at a migrant outpatient clinic.	Migrants residing in seven centres for refugees and asylum seekers (*n* = 330)	(1) HBV screening rate: 30% (99/330); (2) Rate of linkage to care: 0% (0/6). (3) HBV vaccination rate: 9.1% (9/99)	**Factors influencing retention into HBV care** **Length of stay in host countries**. HBsAg positive migrants who were residing in Italy for more than six months were less likely to be lost to follow-up care or treatment compared with those residing in Italy less than 6 months (2/10 vs 9/13; *P* = 0.02).
Nyamathi et al. 2009 [31], USA	A quantitative descriptive study embedded in an RCT conducted at shelters, drug treatment recovery sites, and outdoor locations. HBV vaccine was offered for free in the study.	HBV-negative homeless adults (*n* = 865)	HBV vaccination completion rates: 67.7% (225/332), 60.9% (171/281), and 54% (136/252) in NCMIT, SIT, and SI groups, respectively.	**Factors influencing completion of hepatitis B vaccination** (1) **Age**. Age increased the odds of homeless adults completing HBV vaccine series (*aOR* = 1.03, 95% *CI*: 1.03‒1.07, *P* = 0.001). (2) **Sex**. Male homeless adults were less likely to complete HBV vaccine series compared to females (*aOR* = 0.58, 95% *CI*: 0.37‒0.90, *P* = 0.016). (3) **Ethnicity**. Compared to African Americans, Caucasian homeless individuals were less likely to complete HBV vaccine series (*aOR* = 0.41, 95% *CI*: 0.21‒0.79, *P* = 0.008). (4) **Self-rated health status**. Homeless adults who perceived having a fair or poor health status were more likely to complete HBV vaccine series (aOR = 1.58, 95% *CI*: 1.08‒2.31, *P* = 0.019). (4) **Recent attendance to self-help drug treatment programmes**. Recent attendance to self-help drug treatment programmes were less likely to complete HBV vaccine series compared to non-attendees (*aOR* = 0.66, 95% *CI*: 0.48‒0.90, *P* = 0.010). (6) **Factors examined without showing a significant association**. Being partnered.
Nyamathi et al. 2015 [32], USA	A quantitative descriptive study embedded in an RCT conducted at a service centre for marginalised populations. HBV vaccine was offered for free in the study.	Homeless male parolees from prison or jail (*n* = 345)	HBV vaccination completion rates: 75.4% (86/114), 71.8% (84/117), and 71.9% (82/114) in NCM-PC, peer coaching, and usual care (20-min health promotion counselling) groups, respectively	**Factors influencing non-completion of HBV vaccine series** (1) **Race**. Homeless male parolees who were Asian/Pacific Islanders had higher odds of HBV vaccine non-completion compared to that of Caucasians (*aOR* = 5.86, 95% *CI*: 1.23‒27.92, *P* = 0.03). (2) **Social**. Social support was positively associated with HBV vaccine non-completion (*aOR* = 1.10, 95% *CI*: 1.00‒1.21, *P* = 0.04), while having six or more friends was negatively associated with HBV vaccine non-completion (*aOR* = 0.46, 95% *CI*: 0.22‒0.95, *P* = 0.04). (3) **Situational issues**. Post realignment was positively associated with HBV vaccine non-completion (*aOR* = 2.21, 95% *CI*: 1.19‒4.09, *P* = 0.01), whereas residential drug treatment (at least 90 days) was negatively associated (*aOR* = 0.06, 95% *CI*: 0.03‒0.13, *P* = 0.001). (4) **Cocaine and injection drug utilisation**. Homeless male parolees who used cocaine were less likely to have incomplete HBV vaccination (*aOR* = 0.34, 95% *CI*: 0.16‒0.73, *P* = 0.006), whereas those who had ever injected drugs were more likely to have incomplete HBV vaccination (*aOR* = 2.19, 95% *CI*: 1.07‒4.47, *P* = 0.03). (5) **Hostility**. Homeless male parolees who had higher levels of hostility were more likely to have HBV vaccine noncompletion (*aOR* = 2.24, 95% *CI*: 1.06‒4.73, *P* = 0.04). (6) **Psychiatric hospitalisation**. Homeless male parolees with a history of psychiatric hospitalisation were more likely not to complete HBV vaccine series (*aOR* = 2.58, 95% *CI*: 1.22‒5.46, *P* = 0.01). (7) **Factors examined without showing a significant association**. Received intervention types, and having any alcohol treatment.
Plugee et al. 2007 [33], UK	A cross-sectional study conducted at two women’s prisons. Accelerated HBV vaccine schedule (0, 7, and 21 days) have been introduced for all prisoners in England and Wales.	Women prisoners (*n* = 487)	HBV vaccination rate: 46.8% (228/487) and 27.3% (133/487) received one and three or more doses, respectively	**Factors influencing HBV vaccination** (1) **Previous imprisonment**. Women prisoners who had previous imprisonment for six months (*aOR* = 2.93, 95% *CI*: 1.23‒6.96, *P* = 0.015) or longer (*aOR* = 12.01, 95% *CI*: 5.53‒26.10, *P* < 0.001) were more likely to have undertaken three-dose HBV vaccination. (2) **Drug injection**. Previous intravenous drug use increased the likelihood of undergoing three-dose HBV vaccination among women prisoners (*aOR* = 2.15, 95% *CI*: 1.24‒3.73, *P* = 0.006). (3) **Factors examined without showing a significant association**. Registration with a general practitioner, contact with drug/alcohol services, previous treatment for sexually transmitted diseases, and exchanging money/goods for sex.
Ranjan et al. 2019 [34], Canada	Secondary data analysis from a cross-sectional study.	Female sex workers (*n* = 855)	(1) Lifetime HBV vaccination: 68.3% (584/855) (2) HBV vaccination in the last 6 months: 15.8% (111/702)	**Factors influencing recent or lifetime HBV vaccination** (1) **Immigration status.** Immigration to Canada reduced the odds of self-reported lifetime HBV vaccination among female sex workers (*aOR* = 0.50, 95% *CI*: 0.32‒0.78,* P* = 0.002). (2) **Injection drug use.** A history of injection drug use enhanced the odds of self-reported lifetime HBV vaccination among female sex workers (*aOR* = 1.88, 95% *CI*: 1.27‒2.78, *P* = 0.002). (3) **HIV/STI testing status.** A history of HIV testing (*aOR* = 1.94, 95% *CI*: 1.14‒3.29, *P* = 0.014) and a recent STI testing (*aOR* = 2.95, 95% *CI*: 1.99‒4.39, *P* < 0.001) increased the likelihood of female sex workers having lifetime and recent HBV vaccination in the last six months, respectively. (4) **HIV seropositivity.** HIV seropositivity increased the likelihood of self-reported HBV vaccination in the last six months among female sexual workers (*aOR* = 1.93, 95% *CI*: 1.26‒2.97, *P* = 0.003). (5) **Factors examined without showing a significant correlation.** Age.
Reynolds et al. 2016 [35], USA	A cross sectional study conducted at a community health centre where free HBV vaccination was offered to adults.	Low-income minority adults (*n* = 625)	HBV vaccination rate: 27.6% (173/627), 41.7% (261/625), and 30.5% (191/625) received one, two, and three doses respectively.	**Factors influencing HBV vaccination** (1) **Sex**. Male sex was inversely associated with receiving a higher number of HBV vaccine doses (*β*= -0.088, *P* = 0.021). (2) **Ever had syphilis**. Having ever had syphilis was inversely associated with a higher number of HBV vaccine doses (*β*= -0.283, *P* = 0.002). (3) **Coping strategies**. A higher problem-solving score was positively associated with a higher number of HBV vaccine doses (*β*= 0.017, *P* = 0.001), whereas seeking social support was negatively associated (*β* = -0.008, *P* = 0.016). (4) **Negative emotions**. A higher level of negative emotions was negatively associated with a higher number of HBV vaccine doses (*β*= -0.007,* P* = 0.043). (5) **Factors examined without showing a significant association**. Physical activity level.
Sahajian et al. 2010 [16], France	A quantitative descriptive study embedded in an RCT conducted at shelters.	Underprivileged people living in shelters (*n* = 1276)	The HBV screening completion rate was 1.5% (12/811), 42.8% (95/222), and 59.7% (145/243) for participants receiving no intervention, group information plus screening referral or in-site screening, respectively	**Factors influencing HBV screening completion** (1) **Age.** Among underprivileged people who did not receive any intervention, 18-30-year-olds were independently associated with HBV screening completion (*aOR* = 5.7, 95% *CI*: 1.6‒20.2, *P* < 0.001). (2) **Shelter capacity.** Among underprivileged people who did not receive any intervention, shelter capacity > 160 was positively associated with HBV screening completion (*aOR* = 6.4, 95% *CI*: 1.3‒42.4, *P* = 0.01). (3) **Factors examined without showing a significant association.** Shelter distance and professional activity.
Santilli et al. 2018 [36], France	Ethnographic research conducted at health centres.	Irregular migrants, mainly asylum seekers, infected with HBV (*n* = 30)	N.A.	**Barriers and facilitators to HBV care** (1) **Public policies**. Having a residence permit for medical reasons permits free access to healthcare and facilitates irregular migrants undertake regular HBV treatments in hospitals in Italy and France. Humanitarian health centres also help manage irregular migrants with HBV. In contrast, individuals granted refugee status had to pay for healthcare, including HBV screening. (2) **Immunisation and physician recommendation**. Although HBV-testing was made free owing to the National Action Plan 2009‒2012 in France, it was distributed unevenly and depended on physician’s decision. (3) **Working and living status**. Working illegally, being busy with seasonal work, and living in a different region also precluded irregular migrants from accessing medical treatment after being diagnosed with HBV.
Shahid et al. 2013 [37], Pakistan	A cross-sectional study conducted at roadside barber shops.	Roadside barbers (*n* = 103) and clients (*n* = 103) with low socio-economic status	HBV vaccination rate: 1.9 % (2/103) and 6.8% (7/103) among roadside barbers and their clients, respectively	**Barriers to HBV vaccination** Lack of awareness of the HBV vaccine and the cost of the vaccine were the main barriers to HBV vaccination expressed by roadside barbers and their clients.
Stein et al. 2010 [38], USA	Secondary data analysis of an RCT conducted at homeless shelters, residential substance abuse recovery sites, and outdoor locations. HBV vaccine was offered for free in the study.	Homeless adults (*n* = 331)	HBV vaccination completion rates: 67.7% (224/331)	**Factors influencing HBV vaccination completion** (1) **Prison history.** Not having a previous history of prison significantly predict HBV vaccination completion (*r* = -0.12). (2) **Ethnicity.** African-American ethnicity predicted completion of HBV vaccine series (*r* = 0.10). (3) **Coping strategies.** Positive coping strategies associated with HBV vaccine series (*r* = 0.12). (4) **Social support.** A higher level of social support was positively associated with HBV vaccination completion (*r* = 0.09). (5) **Self-rated health status.** A poor health status significantly predicts HBV vaccine completion (*r* = 0.26). (6) **Self-efficacy.** Higher self-efficacy was positively associated with HBV vaccine completion (*r* = 0.14). (7) **Factors examined without showing a significant association.** Age, needle sharing, unprotected sex, sex, education, knowledge, and intention.
Weis-Torres et al. 2020 [39], Brazil	A cross-sectional study conducted at dumping ground and recycling cooperatives.	Recyclable waste collectors (*n* = 278)	HBV vaccination rate: 26.3% (73/278)	**Factors influencing HBV vaccination** (1) **Age**. Recyclable waste collectors aged 18-25 years were more likely to have HBV vaccination (isolated anti-HBsAb ≥ 10 mIU/mL) than those aged ≥ 26 years (*aOR* = 4.63, 95% *CI*: 2.39‒8.98, *P* < 0.001). (2) **Education**. Recyclable waste collectors with more than nine years of education were more likely to be vaccinated against HBV than those with one to eight years (*aOR* = 1.98, 95% *CI*: 1.07‒3.65, *P* = 0.029). (3) **Knowledge of HBV transmission**. Recyclable waste collectors who have higher knowledge of HBV transmission have higher odds of HBV vaccination (*aOR* = 3.08, 95% *CI*: 1.48‒6.42, *P* = 0.003). (4) **Factors examined without showing a significant association**. Tattoos.
Wong et al. 2018 [40], USA	A prospective cohort study at a diverse, underserved safety-net hospital.	Those underserved and living at/below poverty level (*n* = 693)	(1) HBV screening rate: 25.1% (174/693) (2) Awareness of prior HBV screening: 18.4% (32/174)	**Factors influencing HBV screening** (1) **Race**. Asian (19% vs. 44.4%,* P* < 0.001) and Hispanic (20% vs. 44.4%, *P* < 0.001) individuals were less likely to have previous HBV screening compared to Caucasian individuals. (2) **Birth place**. US-born individuals were more likely to have previous HBV screening compared to those foreign-born (39.1% vs. 22%,* P* < 0.001). (3) **Factors examined without showing a significant association**. Sex, history of intravenous drug use, HIV status.
Wouters et al. 2007 [41], Belgium	Secondary data analysis of an RCT conducted at seven health centres. HBV vaccinations were offered for free at the health centres.	Non-immune sex workers (*n* = 615)	Three-dose HBV vaccination rate: 56.6% (348/615)	**Factors influencing compliance to the HBV vaccination schedule** **Age**. Sex workers aged > 40 years had higher odds of compliance to the three-dose HBV vaccination schedule compared to those aged < 21 years (*OR* = 3.02, 95% *CI*: 1.23‒7.40, *P* = 0.016).
Xiang et al. 2019 [42], China	A cross-sectional study conducted in Chongqing.	Rural migrant workers (*n* = 1,574)	HBV vaccination rate: 49.1% (773/1,574)	**Reasons for not having HBV vaccination** (1) Never heard of HBV vaccine (40%, 206/516). (2) Not knowing where to receive HBV vaccination (31.1%, 160/516). (3) Distrust of HBV vaccine regarding its efficacy and safety (16.3%, 84/516). (4) Inconvenience or inaccessibility to HBV vaccination services (10.7%, 55/516). (5) Family or friends have not been vaccinated against HBV (10.7%, 55/516). (6) Excessive out-of-pocket expenses required (8.2%, 42/516).
				**Factors influencing HBV vaccination** (1) **Age**. Age negatively predicted HBV vaccination among rural migrant workers (*aOR* = 0.98, 95% *CI*: 0.96‒0.99). (3) **Educational level**. Rural migrant workers who were educated at junior middle (*aOR*= 1.85, 95% *CI*: 1.24‒2.74), high school (*aOR* = 1.94, 95% *CI*: 1.25‒3.00), college and above (*aOR* = 2.58, 95% *CI*: 1.57‒4.24) were more likely to have received HBV vaccine than those educated at primary school level. (3) **HBV knowledge**. Rural migrant workers who had higher HBV knowledge were more likely to be vaccinated against HBV (*aOR* = 1.17, 95% *CI*: 1.13‒1.22). (4) **Perceived HBV risk**. A higher perceived HBV risk increased the odds of rural migrant workers undertaking HBV vaccine (*aOR* = 1.40, 95% *CI*: 1.11‒1.75). (5) **Condom usage**. Using condoms less than five times in the past half year increased the likelihood of rural migrant workers receiving HBV vaccine than those not (*aOR* = 1.35, 95% *CI*: 1.07‒1.70). (6) **Factors examined without showing a significant association**. Hometown, ethnicity, years being a migrant worker, accommodation, job position, working hours per day, monthly personal income, cigarette consumption, and having commercial sex.

*adjPR*, adjusted prevalence ratios; *aOR*, adjusted odds ration; *CI*, confidence interval; DNA, deoxyribonucleic acid; HBeAg, hepatitis B e antigen; HIV, human immunodeficiency virus; *IRR*, incidence rate ratio; NCMIT, nurse case management with incentives and tracking (including targeted hepatitis B education, nurse case management, client tracking by outreach workers, incentives for receiving HBV vaccine with $5 for each dose, and a local community resource guide); NCM-PC, peer coaching-nurse case management (including peer coaching focusing on effective coping skills and self-esteem building and nurse management focusing on health promotion, vaccination compliance, and reduction of risky sexual behaviours); RCT, randomised controlled trial; SI, standard intervention (including standard targeted hepatitis education plus incentives and tracking only); SIT, standard incentives and tracking (including standard targeted hepatitis education plus incentives and tracking); USA, the United States of America

### Critical appraisal

With critical appraisal ratings ranging from 43% to 100%, all included quantitative (*n* = 19) and qualitative (*n* = 2) studies attained moderate-to-excellent methodological quality (Supplementary material 2).

The research questions were clearly stated in all the included quantitative studies, and the collected data were adequate to address the study objectives. However, the sampling strategy in five included studies may have been inappropriate to address the research question or recruit a representative sample, as convenience sampling was applied [37, 39, 41] or relevant information (e.g. participant recruitment and inclusion criteria) was not clearly stated [30, 35]. Seven studies either collected self-reported HBV vaccination status [23, 28, 33, 34, 42] or did not report details regarding the reliability or validity of the applied measurements [31, 37]. In addition, one study had a high risk of nonresponse bias (44%) [26] and six studies did not specify the number of nonrespondents [28, 34, 35, 37, 38, 40]. In contrast, most studies had applied appropriate statistical methods, such as multivariate models, to analyse the factors influencing HBV care among the marginalised poor.

Regarding qualitative studies, one was rated as having excellent methodological quality [25] while the other had a quality assessment score of 57%, since findings were not consolidated by participant quotes, and a lack of coherence existed between data collection, analysis, and interpretations [36].

### HBV healthcare utilisation among marginalised poor populations

**Fig. 2:**
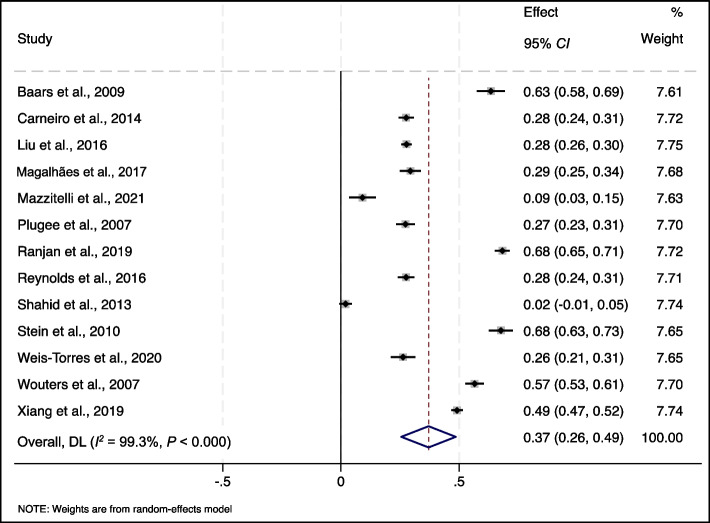
Forest plot of the pooled at least one-dose hepatitis B virus (HBV) vaccination rates among marginalize poor populations, applying a random-effect model

Thirteen studies reported rates of at least one HBV vaccination dose among marginalised poor populations. A meta-analysis of the thirteen studies showed a 37% pooled uptake rate (95% *CI*: 0.26‒0.49, *P* < 0.001, *I*^*2*^ = 99.3%) [23, 24, 27, 28, 30, 33–35, 37–39, 41, 42] (Fig. 2). Sensitivity analysis showed that the pooled rates of at least one HBV vaccination dose were not affected by the removal of any individual study (Supplementary material 2). Three studies reported HBV screening rates ranging from 1.5% to 27.5% among immigrants with low income [26], the underprivileged [16], and poor populations [40]. The rates of linkage-to-care and HBV treatment after HBV diagnoses among immigrants with low income were 0% [30] and 15.9% [26], respectively.

### Factors influencing access to HBV healthcare services among marginalised poor populations

**Fig. 3:**
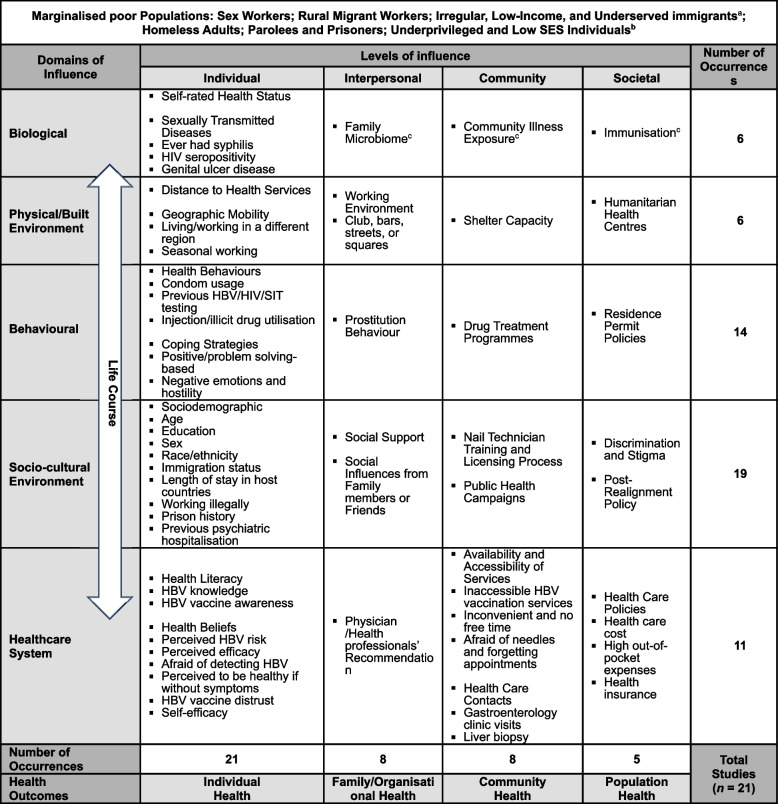
Factors influencing hepatitis B virus (HBV) healthcare access among marginalised poor populations mapped onto the National Institute on Minority Health and Disparities Research Framework. *Note*: ^a^Immigrants with low income, migrants residing in centres for refugees and asylum seekers, irregular migrants (mainly asylum seekers), minority adults with low income, Vietnamese nail salon workers from underserved communities; ^b^Underprivileged people living in shelters, roadside barbers and clients, collectors of recyclable waste, underserved, and residing below poverty levels; ^c^Factors included in the National Institute on Minority Health and Health Disparity research framework but were not reported in the included studies of this review

A total of 51 influencing factors were identified and mapped onto the NIMHD research framework (Fig. 3). The socio-cultural environment domain influencing factors were the most commonly reported (*n* = 19), followed by behavioural (*n* = 14) and healthcare system domains (*n* = 11). Each included study reported individual-level influencing factors. The details are interpreted in Fig. 3.

### Biological domain

Regarding biological influencing factors, perceived poor health status [31, 38] and a history of sexually transmitted diseases, including syphilis [35] and human immunodeficiency virus [26, 34], were associated with HBV vaccine completion and HBV treatment receipt among marginalised poor populations, including homeless adults, minority adults with low income, and female sex workers. Poor health status and experience of disease may increase awareness of health issues and care-seeking, including HBV vaccination and treatment.

### Physical/built environment domain

At the individual level, geographic mobility, including moving to a different region and engaging in seasonal work, disrupted the HBV care continuum and was the main barrier to HBV vaccination and treatment among marginalised poor populations (including female sex workers and irregular migrants) [23, 28, 36].

At the interpersonal level, it was noteworthy that female sex workers employed in erotic show houses and clubs presented higher odds of not completing the HBV vaccination schedule compared to those working on streets, massage parlours, brothels, bars, or squares [24, 28]. This association might be partially attributable to the higher earnings and turnover rates among women working in erotic show houses and clubs, which may induce additional barriers to vaccine completion, such as lack of time [28].

At the community and societal levels, high-capacity shelters (capacity > 160) increased the likelihood of underprivileged individuals completing HBV screening by approximately 6.4 times compared to those living in low-capacity shelters (capacity < 160) [16]. Nevertheless, those living in low-capacity shelters might have closer contact and higher risks of HBV contamination [16], necessitating additional interventions. In addition, humanitarian health centres also facilitate HBV treatment and management, especially for irregular migrants with HBV [36].

### Behavioural domain

**Fig. 4:**
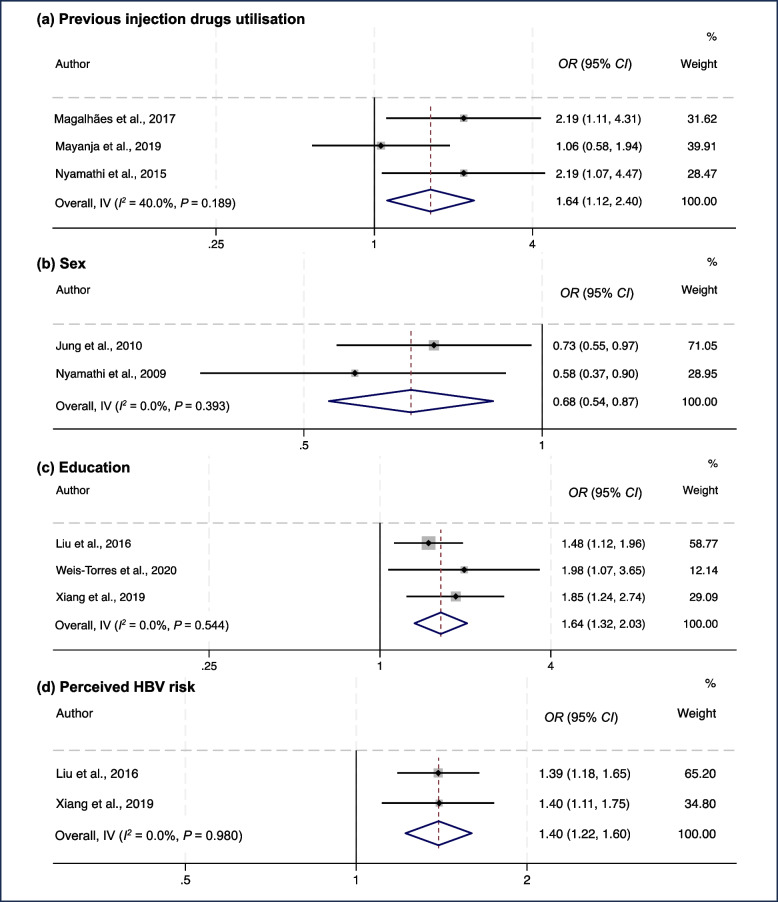
Forest plot of the pooled odds ratio (*OR*) of influencing factors, including previous injection drug use (**a**), sex (**b**), education (**c**), and perceived HBV risk (**d**), on HBV vaccination among marginalised poor populations, applying fixed-effects models

At the individual level, health behaviours and coping strategies were associated with HBV healthcare access among marginalised poor populations. Compared to inconsistent utilisation, consistent condom use was identified as a risk factor for non-completion of HBV vaccination doses among female sex workers and rural migrant workers [29, 42]. This effect may be associated with the perception that condom use could protect against HBV, leading to a lower perceived need for vaccination. Conversely, previous testing of HBV or sexually transmitted diseases were consistently reported as a facilitator for HBV vaccination and treatment among immigrants with low income and female sex workers [26, 34]. However, conflicting results regarding the effects of illicit drug use have also been reported. Two studies [33, 34] found significantly higher odds of HBV vaccination among female sex workers and female prisoners with a history of injection drug use. Conversely, previous illicit or injection drugs use predicted non-completion of HBV vaccination in the combined effects in other three studies conducted among female sex workers and homeless male parolees (*OR* = 1.64, 95% *CI*: 1.12‒2.40, *I*^*2*^ = 40%,* P* = 0.189) (Fig. 4a) [28, 29, 32].

Positive coping strategies, including problem-solving focus, were positively associated with completing the HBV vaccination series [35, 38]. Conversely, negative emotions (e.g. hostility) were negatively associated with completion among minorities with low income, homeless adults, and male parolees [32, 35].

At the interpersonal, community, and societal levels, female sex workers with less than one year of prostitution experience were less likely to complete HBV vaccination doses [24], suggesting a lower perceived risk of HBV infection and vaccination need compared to those with more years of prostitution [23]. Additionally, homeless adults with over 90 days of residential drug treatment were more likely to complete the HBV vaccination series than their counterparts who received less than 90 days of treatment [32]. By contrast, those attending self-help drug treatment programmes were more likely not to complete the vaccination programme compared to non-attendees [31]. Moreover, irregular migrants in Italy and France with a residence permit for medical reasons had access to free healthcare, facilitating regular HBV treatment [36].

### Socio-cultural environment domain

Sociodemographic factors, including sex, race, immigration, and prison history, were common individual-level influencing factors. Among immigrants with low income and homeless adults, being male consistently predicted non-completion of the HBV vaccine series, with a combined *OR* value of 0.68 (95% *CI*: 0.54‒0.87, *I*^*2*^ = 0%,* P* = 0.393) (Fig. 4b) [26, 31]. Furthermore, African Americans [31, 38] and Asian/Pacific Islanders [32] were more likely to complete HBV vaccine doses than Caucasians. Moreover, immigration status [34, 40], residing in host countries for less than six months [30], working illegally [36], and previous psychiatric hospitalisation [32] were negatively associated with HBV screening, lifetime vaccination, treatment, and follow-up care among underserved individuals, homeless parolees, female sex workers, and irregular migrants, such as refugees and asylum seekers. Additionally, no current or previous prison history was associated with HBV vaccination completion among homeless adults and female sex workers [33, 38].

However, discrepancies existed between studies regarding certain demographic variables, including age, educational level, and HBV healthcare utilisation. Four studies showed that age was negatively associated with HBV screening and vaccination among rural migrant workers, collectors of recyclable waste, and underprivileged people [16, 27, 39, 42]. This may be partially attributed to the national HBV vaccination plan for newborns and infants since the 1990s; younger individuals (e.g. aged 18 to 30 years) were more likely to fall within the required age for vaccination when the policy was introduced [42]. This contrasts with four other studies, in which a positive [29, 31, 41] or insignificant [38] correlation between age and HBV vaccination was observed. Similarly, a meta-analysis of three studies with *OR* values showed that a higher education level was associated with higher odds of rural migrant workers and collectors of recyclable waste undergoing HBV vaccination compared to those with a primary educational level or those with less than nine years of education (*OR* = 1.64, 95% *CI*: 1.32‒2.03, *I*^*2*^ = 0%, *P* = 0.544) (Fig. 4c) [27, 39, 42]. However, two studies showed no correlation or a reverse correlation between educational level and HBV vaccine completion [24, 38].

At interpersonal level, studies have yielded mixed results regarding whether social support was positively associated with HBV vaccination completion among homeless adults and parolees [32, 38]. However, the vaccination status of family members and friends against HBV may have influenced rural migrant workers to receive the vaccination [42].

At the community and societal levels, community professional training programmes, public health campaigns, discrimination and stigma against HBV, and situational issues such as the prison realignment policy were also significant factors influencing HBV healthcare access. For example, a nail technician training programme for Vietnamese nail salon workers did not convey specific information regarding HBV and even spread misinformation by suggesting that HBV could be prevented by wearing masks [25]. In addition, owing to the knowledge gaps in HBV transmission and fears of infection, HBV discrimination and stigma (e.g. avoiding close contact with HBV-infected individuals) still exist within the community, posing another barrier to HBV care [25]. Vietnamese nail salon workers expressed that public health campaigns, including leaflets and brochures regarding HBV in nail salons, churches, and other Vietnamese communities, would be helpful in increasing HBV awareness and facilitate access to HBV vaccination [25]. Finally, among homeless male parolees, those released after the prison realignment policy were approximately 2.21 times more likely not to complete HBV vaccination compared to those released before realignment [32].

### Healthcare system

At the individual level, health literacy and beliefs regarding HBV were associated with HBV healthcare utilisation among marginalised poor populations. Limited health literacy, including not having heard of HBV and misperceptions about its transmission, symptoms, and prevention strategies [23, 25], and unawareness of the HBV vaccine, its costs, and service access [42] precluded Vietnamese nail salon workers, rural migrant workers, female sex workers, roadside barbers, and their clients from seeking HBV vaccination. Conversely, higher levels of HBV knowledge (including knowledge of HBV transmission, vaccination, and screening tests) increased the likelihood of rural migrant workers [42] and collectors of recyclable waste [39] undergoing HBV vaccination by up to 3 times.

Regarding health beliefs, a meta-analysis of two studies [27, 42] showed that perceived HBV risk and vulnerability significantly increased the odds of rural migrant workers undergoing HBV vaccination (*OR* = 1.40, 95% *CI*: 1.22‒1.60, *I*^*2*^ = 0%, *P* = 0.980) (Fig. 4d). The perceived efficacy [27] of the HBV vaccine was also associated with HBV screening and vaccination among immigrants with low income and rural migrant workers. In contrast, fear of detecting HBV, the perception of being healthy if asymptomatic [25], and HBV vaccine distrust [42] precluded marginalised poor populations from seeking HBV screening, vaccination, and care. However, the perceived severity of HBV was not significantly associated with HBV vaccination [27] and conflicting results were found regarding whether self-efficacy was positively associated with HBV vaccination completion among rural migrant workers and homeless adults [27, 38].

At the interpersonal level, physician recommendations were essential for Vietnamese nail salon workers and irregular migrants to undergo HBV screening [25, 36]. Otherwise, they might assume that a normal blood test would include HBV testing and perceive that they are not infected if the results were normal [25]. Moreover, receiving information about HBV vaccination programmes from healthcare professionals increased the odds of sex workers receiving at least one HBV vaccine dose by up to 4.27 times [23].

At the community level, inaccessibility [42] or inconvenience of HBV care services, including no free time and busy working hours [23, 25, 28], were negatively associated with HBV vaccination and care. According to Baars et al. [23], fear of needles and forgetting appointments also discouraged female sex workers from undergoing HBV vaccination [23]. Conversely, more visits to gastroenterology clinics and prior liver biopsy procedures increased the odds of immigrants with low income undergoing HBV screening and treatment by 2.6 and 5.4 times, respectively [26].

At the societal level, the cost of HBV vaccine, high out-of-pocket expenses, and lack of insurance were the main barriers to HBV vaccination and care among Vietnamese nail salon workers [25], rural migrant workers [42], and roadside barbers and their clients [37].

## Discussion

This mixed-method systematic review represents the comprehensive synthesis of HBV healthcare utilisation and its influencing factors among marginalised poor populations. The synthesis of 21 studies highlights a situation in which the pooled rate of HBV vaccination is merely 37%, and the rates for HBV screening, treatment, and linkage-to-care are less than 27.5%. Guided by the NIMHD research framework, 51 influencing factors were identified across biological (e.g. self-rated health status), physical/built environment (e.g. geographic mobility), behavioural (e.g. hostile coping strategy), socio-cultural environment (e.g. immigration, discrimination, and stigma), and healthcare system (e.g. health literacy and beliefs about HBV, availability, and accessibility of services) domains. These insights could inform the development of health education, HBV tracking and management systems, tailored community outreach programmes, and human rights-based policy frameworks to improve HBV healthcare access, ultimately paving the way for HBV elimination among marginalised poor populations.

The utilisation of HBV healthcare services among marginalised poor populations has been inadequately documented in the literature, with only 21 relevant studies included in this review. Reaching these populations is challenging due to logistical constraints, socioeconomic instability, and the lack of a robust healthcare infrastructure, all of which significantly impede systematic data collection and reporting [8]. According to three included studies [16, 26, 40], the rates of HBV screening and treatment among these populations were exceedingly low, with both less than 27.5% and 15.9%, respectively. These figures fall alarmingly short of the World Health Organization’s targets for HBV elimination, which aim for 90% diagnosis and 80% treatment coverage by 2030 [5]. Moreover, this review found that the pooled HBV vaccination rate with at least one dose was only 37%. These findings are consistent with those of a previous systematic review that revealed that referral, follow-up, and initiation of care for infectious diseases (hepatitis C virus and human immunodeficiency virus) among marginalised poor populations were below 30% [43]. These data underscore the disparities in HBV protection, diagnosis, and subsequent linkage-to-care among marginalised poor populations, all which potentially exacerbate the prevalence of HBV infection.

In the physical/built environment domain, geographical mobility emerged as a major obstacle to HBV vaccination and treatment among marginalised poor populations, especially migrant workers, irregular migrants, and female sex workers. Consistent with previous reviews, geographical mobility precluded marginalised migrant labourers from accessing health and vaccination services, leaving them more vulnerable to infectious diseases [44]. These groups often move across regions because of their illegal immigration status, seasonal working, or financial constraints [36]. The transient nature of these populations, along with their unfamiliarity with health systems in new regions, might obstruct their access to healthcare services, impede the completion of the HBV vaccine series, and lead to discontinuity in HBV care [23, 28]. Mobility also makes it difficult to track HBV vaccination, treatment, and care requirements. This highlights that healthcare delivery and policy reform should be tailored to the circumstances of mobility among marginalised poor populations.

Regarding the behavioural domain, this review highlights the influence of negative emotional coping strategies, specifically hostility, as a barrier to HBV vaccine uptake. Consistent with previous literature, hostility is common among marginalised poor populations and creates a significant obstacle to healthcare [45]. Individuals exhibiting hostility demonstrate traits of irritation, cynicism, and mistrust, making them resistant to conforming to societal norms and complying with healthcare instructions from perceived authoritative figures, thereby complicating efforts to promote HBV vaccination [45, 46]. The coping strategy of hostility represents a multifaceted interplay of psychological, social, cultural, and policy dynamics that perpetuate negative emotions within marginalised poor populations [47]. Severe depression, societal stereotypes (e.g. associating poverty with laziness, uncleanliness, and criminality), and discriminatory policies aimed at marginalised groups exacerbate feelings of hostility and alienation [48, 49]. Future studies should further identify and address the complex factors that contribute to hostility among marginalised poor populations. Holistic approaches and collaborative efforts are suggested to mitigate hostility and enhance HBV vaccination among marginalised poor populations.

In the socio-cultural environment domain, migration status significantly impacts the accessibility of HBV healthcare for marginalised poor populations. Consistent with previous research [15], immigration presents a dilemma for healthcare access, particularly among immigrant sex workers [34] and irregular migrants [36]. Illegal immigration status, absence of a residence permit, unauthorised employment, fear of authorities, and discrimination for their occupations (e.g. sex work) make their access to healthcare extremely difficult [50, 51]. Additionally, the scarcity of healthcare personnel and limited health resources allocated for irregular immigrants may further exacerbate the deficit in HBV healthcare provision [52]. Although few countries (e.g. the Netherlands and Brazil) have launched HBV vaccination programmes accessible to migrant sex workers, access to such care remains hindered by structural obstacles, including mobility and difficulties with public transportation [34]. More importantly, disruptions in federal healthcare systems obstruct HBV treatment and care for irregular immigrants relocating within the country for work [36]. Consequently, the incidence of invisible migrants infected with HBV is heightened [53]. It is imperative for governments and healthcare systems to intensify their focus on marginalised immigrant groups, promote HBV screening and vaccination services upon their arrival in host countries, and implement targeted outreach programmes to address the complex challenges in HBV treatment and care faced by these vulnerable populations.

In the healthcare system domain, significant knowledge gaps exist regarding HBV among marginalised poor populations, affecting their health beliefs and impeding their engagement with HBV screening and vaccination services [23, 25, 39, 42]. Consistent with previous research [54], many lack comprehension of HBV, including its symptoms, transmission modes, and the preventive benefits of HBV vaccination. Some individuals hold misperceptions, believing that HBV spreads through food and water consumption, or mosquito bites [25]. Such misperceptions can exacerbate fear and stigmatisation linked to HBV, leading to hesitancy in seeking screening services due to apprehensions about potential stigma following a positive diagnosis [55]. Additionally, some believe that HBV transmission occurs only within families, which diminishes their perceived risk and susceptibility to HBV infection, consequently decreasing their likelihood of seeking HBV screening and vaccination [25]. Consistent with the findings of this review, previous studies have also shown that marginalised poor populations (e.g. farmers and migrants with low income) exhibit a limited understanding of HBV symptoms and often neglect or delay seeking HBV screening or treatment, even when displaying overt HBV symptoms, such as scleral jaundice [56]. Furthermore, marginalised poor populations are unaware of the existence of the HBV vaccine, lack information on where to access vaccination services, and have doubts regarding its safety and efficacy [42, 54]. Compounded by lower levels of formal education, the population encounters challenges in accessing, comprehending, and utilising information related to HBV prevention and control [57]. It is imperative to prioritise targeted HBV education and awareness campaigns and promote immunisation outreach initiatives among marginalised poor communities.

More importantly, marginalised poor populations usually have low income and uninsured positions (e.g. roadside barbers and collectors of recyclable waste), and healthcare costs, lack of insurance coverage, and high rates of out-of-pocket payments become major deterrents to HBV healthcare access [25, 42]. This echoes the findings of previous studies that marginalised poor populations have higher uninsured rates and are inaccessible to affordable healthcare services [58, 59]. They might struggle to obtain basic needs (e.g. food, water, and housing), let alone access non-emergent preventive healthcare measures, such as HBV screening and vaccination [56, 59]. Additionally, some marginalised poor populations, including nail technicians, female sex workers, and rural migrant workers, face additional challenges due to long working hours, often extending to seven days a week, making timely scheduling of HBV healthcare difficult [23, 28]. Disparities in healthcare service access may ultimately lead to delayed HBV diagnosis, poor treatment, and diminished health outcomes, which, in turn, increase healthcare costs and financial burdens among marginalised poor populations and their families. This perpetuates a vicious cycle that further impedes access to HBV healthcare services. A more equitable health system should be established to guarantee healthcare coverage, facilitate service access, and empower supporting organisations to reduce HBV disparities among marginalised, poor populations.

This review has several limitations. First, although there was no restriction on the language of publications, this review only searched English and Chinese language databases, potentially missing relevant studies in other languages. Second, this review did not search for gray literature, including news, policy statements, and discussion forums, which could have provided valuable insights into healthcare utilisation among marginalised poor populations. Third, heterogeneity was observed in the pooled analysis of the HBV vaccination rate, which could stem from variations in study design and populations. This may have decreased the certainty of the synthesised evidence. Fourth, HBV vaccination status was self-reported in some included studies [23, 28], potentially introducing underestimation or overestimation and biasing the estimated HBV vaccination rate in this review. Finally, the findings of this review should be interpreted with caution, considering the specific contexts of the included studies. Variations in the availability of HBV vaccinations, population demographics, and intervention approaches among the included studies may have affected generalisability of the findings.

Engagement with HBV healthcare among marginalised poor populations is poorly documented. A substantial portion of this population remains undiagnosed and untreated, exacerbating health inequities towards HBV elimination. Future empirical and modelling research is warranted to capture the missing data. Multilevel strategies addressing the social determinants that hinder HBV healthcare access are also suggested to narrow these inequities.

At the individual level, tailored health education is necessary to dispel misconceptions regarding HBV infection, including its symptoms, transmission routes, and preventive and treatment measures. To accommodate the low educational level of marginalised poor populations, utilising digital media and other technologies to disseminate engaging and easy-to-understand content is suggested to effectively reach and encourage HBV prevention and treatment behaviours. At the healthcare system level, establishing integrated HBV management systems is essential to address the challenges posed by the high geographic mobility of marginalised poor populations. Strategies include implementing mobile health clinics, telemedicine services, and online HBV surveillance platforms that allow continuous tracking and management. Incorporating HBV healthcare services into commonly accessed healthcare settings by marginalised poor populations, such as shelter-based clinics, humanitarian health centres, and drug treatment facilities, can also enhance accessibility and continuity of care. At the community level, bolstering outreach programmes for HBV screening and vaccination in venues frequented by marginalised poor populations, such as nail salons, churches, and ethnic community centres, is essential. Efforts led by community leaders and supported by non-governmental organisations to reduce hostility and stigma surrounding HBV are critical for improving programme reception and adherence. At the policy level, a human rights-based framework is suggested, especially for irregular migrants, to ensure unfettered access to crucial HBV healthcare due to their illegal immigration status. Policy adjustments should also address the right to health insurance, provide complementary HBV screening and vaccination for special groups (such as sex workers and individuals from HBV-prevalent regions), and ensure the availability of ongoing antiviral treatment.

## Conclusions

This systematic review, employing a mixed-method methodology, comprehensively synthesised evidence regarding HBV healthcare utilisation and its influencing factors among marginalised poor populations. It identified significant service access gaps, with rates of HBV screening, treatment, and linkage-to-care alarmingly low among vulnerable populations, ranging from 1.5% to 27.5%. The pooled rate of at least one HBV vaccination dose barely reached 37%. Through the lens of the NIMHD research framework, this review identified 51 influencing factors dispersed across multiple domains: biological (e.g. self-rated health status and sexually transmitted diseases), physical/built environment (e.g. distance to health services and geographic mobility), behavioural (e.g. condom usage and hostile coping strategies), socio-cultural environment (e.g. immigration, discrimination, and stigma), and healthcare system (e.g. health literacy and beliefs about HBV, availability and accessibility of services) domains were identified to influence the HBV healthcare.

To facilitate HBV elimination among vulnerable populations, access to HBV healthcare services—from screening to vaccination, diagnosis, treatment, and follow-up care—should be enhanced. The findings of this review suggest a synergistic approach to counteracting these barriers. This would involve health education initiatives aimed at debunking HBV misperceptions, establishing integrated HBV management systems for continuous tracking and care, tailored outreach programmes geared towards improving screening and vaccination rates, and incorporating a human rights-based policy framework to guarantee unfettered access to essential HBV healthcare for marginalised poor populations.

### Supplementary Information

Supplementary Material 1.

Supplementary Material 2.

## Data Availability

Data availability is not applicable to this review as the data used were synthesised from previous studies.

## Abbreviations

*CI*s: Confidence intervals
HBV: Hepatitis B virus
NIMHD: National Institute on Minority Health and Health Disparity
*OR*s: Odds ratios
PICOS: Participant-Intervention-Comparator-Outcomes-Study design
PRISMA: Preferred Reporting Items for Systematic Reviews and Meta-analyses
