# Supplementary material for: Disparities in hepatitis B virus healthcare service access among marginalised poor populations: a mixed-method systematic review
Source: Infect Dis Poverty. 2024 Aug 9;13:58. doi: 10.1186/s40249-024-01225-0 (PMC11312201; doi:10.1186/s40249-024-01225-0)
Supplement: Supplementary file 1 — Supplementary Material 1. [file 40249_2024_1225_MOESM1_ESM.docx]

**Table 1** Search terms

| **#1**  **Marginalized poor** | exp Vulnerable Populations/ or exp "Health Disparate, Minority and Vulnerable Populations"/ or ((marginalized or marginalised or hard to reach or seldom heard or vulnerabl* or exclude* or segregate* or discriminat* or disadvantage* or minority) adj1 (people or patient* or population* or group* or sample* or participant*)).ab,ti.  OR  exp Ill-Housed Persons/ or exp Homeless Youth/ or (homeless* or home-less or shelter* or unhoused or unstab* hous* or street* or vagabond* or hobo* or drifter*).ab,ti  OR  exp "Transients and Migrants"/ or (Migrant* adj1 (worker* or farmworker*)).ab,ti. or (migrant domestic worker* or migrant farmworker* or migrant construction worker*).ab,ti.  OR  exp Low Socioeconomic Status/ or exp Poverty/ or Poverty Areas/ or ((economic* or social* or financial*) adj1 (disadvantage* or vulnerabl* or low or poor)).ab,ti. or (farmer* or rural or village* or construction worker* or sanitation worker* or unemployed or poor or poverty or underserv* or low-income or low income).ab,ti.  OR  exp Disabled Persons/ or exp Vision Disorders/ or exp Hearing Loss/ or ((Mobility or physical or vision or visual or hearing) adj1 (disab* or impair* or limit* or loss)).ab,ti. or (wheelchair or handicap* or disabled or deaf*).ab,ti. |
| --- | --- |
| **#2**  **Hepatitis B** | exp hepatitis B/ or exp hepatitis B virus/ or (hepatitis B or HBV or hep B or CHB).ab,ti. |
| **#3**  **Screening, vaccination, and linkage to care** | exp Mass screening/ or exp Vaccination/ or exp Vaccination Refusal/ or exp Vaccination Hesitancy/ or Vaccination Coverage/ or exp "Continuity of Patient Care"/ or exp Patient Care/ or (screen* or test* or vaccin* or immun* or linkage to care or linkage-to-care or linkage or linking or care continuum or care or refer* or follow up or follow-up or monitor* or surveillance or examination* or assessment* or visit* or attend* or clinic* or hospital* or hepatolog* or physician* or specialist* or provider* or treatment* or therap* or manag* or counseling or counselling or service* or healthcare or health care).ab,ti. |
| **#4**  **Influencing factors** | (Factor* or influenc* or associat* or correlat* or facilitator* or motivat* or enabl* or promot* or facilitat* or predict* or determin* or barrier* or preclud* or challeng*).ab,ti. |
| **#5** | #1 AND #2 AND #3 AND #4 |

**Table 2** Search record in each included database

| Ovid MEDLINE(R) on May 2, 2023 10:00 PM | | |
| --- | --- | --- |
| #1 | exp hepatitis B/ or exp hepatitis B virus/ or (hepatitis B or HBV or hep B or CHB).ab,ti. | 100593 |
| #2 | exp Mass screening/ or exp Vaccination/ or exp Vaccination Refusal/ or exp Vaccination Hesitancy/ or Vaccination Coverage/ or exp "Continuity of Patient Care"/ or exp Patient Care/ or (screen* or test* or vaccin* or immun* or linkage to care or linkage-to-care or linkage or linking or care continuum or care or refer* or follow up or follow-up or monitor* or surveillance or examination* or assessment* or visit* or attend* or clinic* or hospital* or hepatolog* or physician* or specialist* or provider* or treatment* or therap* or manag* or counseling or counselling or service* or healthcare or health care).ab,ti. | 15807435 |
| #3 | exp Vulnerable Populations/ or exp "Health Disparate, Minority and Vulnerable Populations"/ or ((marginalized or marginalised or hard to reach or seldom heard or vulnerabl* or exclude* or segregate* or discriminat* or disadvantage* or minority) adj1 (people or patient* or population* or group* or sample* or participant*)).ab,ti. | 214632 |
| #4 | exp Ill-Housed Persons/ or exp Homeless Youth/ or (homeless* or home-less or shelter* or unhoused or unstab* hous* or street* or vagabond* or hobo* or drifter*).ab,ti | 40082 |
| #5 | exp "Transients and Migrants"/ or (Migrant* adj1 (worker* or farmworker*)).ab,ti. or (migrant domestic worker* or migrant farmworker* or migrant construction worker*).ab,ti. | 14658 |
| #6 | exp Low Socioeconomic Status/ or exp Poverty/ or Poverty Areas/ or ((economic* or social* or financial*) adj1 (disadvantage* or vulnerabl* or low or poor)).ab,ti. or (farmer* or rural or village* or construction worker* or sanitation worker* or unemployed or poor or poverty or underserv* or low-income or low income).ab,ti. | 872205 |
| #7 | exp Disabled Persons/ or exp Vision Disorders/ or exp Hearing Loss/ or ((Mobility or physical or vision or visual or hearing) adj1 (disab* or impair* or limit* or loss)).ab,ti. or (wheelchair or handicap* or disabled or deaf*).ab,ti. | 322357 |
| #8 | (Factor* or influenc* or associat* or correlat* or facilitator* or motivat* or enabl* or promot* or facilitat* or predict* or determin* or barrier* or preclud* or challeng*).ab,ti. | 12741695 |
| #9 | #3 OR #4 OR #5 OR #6 OR #7 | 1398317 |
| #10 | #1 AND #2 AND #8 AND #9 | 3822 |
| EMBASE on May 4, 2023 | | |
| #1 | exp hepatitis B/ or exp hepatitis B virus/ or (hepatitis B or HBV or hep B or CHB).ab,ti. | 184331 |
| #2 | exp Mass screening/ or exp Vaccination/ or exp Vaccination Refusal/ or exp Vaccination Hesitancy/ or Vaccination Coverage/ or exp "Continuity of Patient Care"/ or exp Patient Care/ or (screen* or test* or vaccin* or immun* or linkage to care or linkage-to-care or linkage or linking or care continuum or care or refer* or follow up or follow-up or monitor* or surveillance or examination* or assessment* or visit* or attend* or clinic* or hospital* or hepatolog* or physician* or specialist* or provider* or treatment* or therap* or manag* or counseling or counselling or service* or healthcare or health care).ab,ti. | 23393279 |
| #3 | exp Vulnerable Populations/ or exp health disparity/ or ((marginalized or marginalised or hard to reach or seldom heard or vulnerabl* or exclude* or segregate* or discriminat* or disadvantage* or minority) adj1 (people or patient* or population* or group* or sample* or participant*)).ab,ti. | 148423 |
| #4 | exp homeless person/ or exp homelessness/ or (homeless* or home-less or shelter* or unhoused or unstab* hous* or street* or vagabond* or hobo* or drifter*).ab,ti. | 57105 |
| #5 | exp migrant worker/ or (Migrant* adj1 (worker* or farmworker*)).ab,ti. or (migrant domestic worker* or migrant farmworker* or migrant construction worker*).ab,ti. | 3354 |
| #6 | exp extreme poverty/ or exp poverty level/ or exp poverty/ or ((economic* or social* or financial*) adj1 (disadvantage* or vulnerabl* or low or poor)).ab,ti. or (farmer* or rural or village* or construction worker* or sanitation worker* or unemployed or poor or poverty or underserv* or low-income or low income).ab,ti. | 1440741 |
| #7 | exp Disabled Persons/ or exp Vision Disorders/ or exp Hearing Loss/ or ((Mobility or physical or vision or visual or hearing) adj1 (disab* or impair* or limit* or loss)).ab,ti. or (wheelchair or handicap* or disabled or deaf*).ab,ti. | 581780 |
| #8 | (Factor* or influenc* or associat* or correlat* or facilitator* or motivat* or enabl* or promot* or facilitat* or predict* or determin* or barrier* or preclud* or challeng*).ab,ti. | 19291128 |
| #9 | 3 or 4 or 5 or 6 or 7 | 2156327 |
| #10 | #1 AND #2 AND #8 AND #9 | 8170 |
| #11 | limit 10 to full text | 2089 |
| #12 | limit 10 to conference abstracts | 2793 |
| #13 | limit 12 to human | 5192 |
| #14 | limit 13 to embase | 4396 |
| Ovid Emcare on May 4, 2023 | | |
| #1 | exp hepatitis B/ or exp hepatitis B virus/ or (hepatitis B or HBV or hep B or CHB).ab,ti. | 21307 |
| #2 | exp screening/ or exp Mass screening/ or exp Vaccination/ or exp Vaccination Refusal/ or exp Vaccination Hesitancy/ or Vaccination Coverage/ or exp Patient Care/ or (screen* or test* or vaccin* or immun* or linkage to care or linkage-to-care or linkage or linking or care continuum or care or refer* or follow up or follow-up or monitor* or surveillance or examination* or assessment* or visit* or attend* or clinic* or hospital* or hepatolog* or physician* or specialist* or provider* or treatment* or therap* or manag* or counseling or counselling or service* or healthcare or health care).ab,ti. | 5181939 |
| #3 | exp Vulnerable Populations/ or exp health disparity/ or ((marginalized or marginalised or hard to reach or seldom heard or vulnerabl* or exclude* or segregate* or discriminat* or disadvantage* or minority) adj1 (people or patient* or population* or group* or sample* or participant*)).ab,ti. | 51391 |
| #4 | exp homeless person/ or exp homeless man/ or exp homeless youth/ or exp homeless woman/ or exp homelessness/ or (homeless* or home-less or shelter* or unhoused or unstab* hous* or street* or vagabond* or hobo* or drifter*).ab,ti. | 23613 |
| #5 | exp migrant worker/ or (Migrant* adj1 (worker* or farmworker*)).ab,ti. or (migrant domestic worker* or migrant farmworker* or migrant construction worker*).ab,ti. | 1492 |
| #6 | exp poor general condition/ or exp extreme poverty/ or exp poverty level/ or exp poverty/ or ((economic* or social* or financial*) adj1 (disadvantage* or vulnerabl* or low or poor)).ab,ti. or (farmer* or rural or village* or construction worker* or sanitation worker* or unemployed or poor or poverty or underserv* or low-income or low income).ab,ti. | 387463 |
| #7 | exp Disabled Persons/ or exp Vision Disorders/ or exp Hearing impairment/ or ((Mobility or physical or vision or visual or hearing) adj1 (disab* or impair* or limit* or loss)).ab,ti. or (wheelchair or handicap* or disabled or deaf*).ab,ti. | 129841 |
| #8 | (Factor* or influenc* or associat* or correlat* or facilitator* or motivat* or enabl* or promot* or facilitat* or predict* or determin* or barrier* or preclud* or challeng*).ab,ti. | 4118658 |
| #9 | #3 or #4 or #5 or #6 or #7 | 567511 |
| #10 | #1 AND #2 AND #8 AND #9 | 1294 |
| Ovid Nursing database on May 4, 2023 | | |
| #1 | exp hepatitis B/ or (hepatitis B or HBV or hep B or CHB).ab,ti. | 237 |
| #2 | exp Health Screening/ or exp Immunization/ or exp Vaccines/ or exp "Continuity of Patient Care"/ or exp Patient Care/ or exp Point-of-Care Testing/ or exp Health Care/ or exp Medical Care/ or (screen* or test* or vaccin* or immun* or linkage to care or linkage-to-care or linkage or linking or care continuum or care or refer* or follow up or follow-up or monitor* or surveillance or examination* or assessment* or visit* or attend* or clinic* or hospital* or hepatolog* or physician* or specialist* or provider* or treatment* or therap* or manag* or counseling or counselling or service* or healthcare or health care).ab,ti. | 202152 |
| #3 | exp Special Populations/ or ((marginalized or marginalised or hard to reach or seldom heard or vulnerabl* or exclude* or segregate* or discriminat* or disadvantage* or minority) adj1 (people or patient* or population* or group* or sample* or participant*)).ab,ti. | 2337 |
| #4 | exp Homeless Shelters/ or exp Homeless Persons/ or exp homelessness/ or (homeless* or home-less or shelter* or unhoused or unstab* hous* or street* or vagabond* or hobo* or drifter*).ab,ti. | 755 |
| #5 | exp "Transients and Migrants"/ or (Migrant* adj1 (worker* or farmworker*)).ab,ti. or (migrant domestic worker* or migrant farmworker* or migrant construction worker*).ab,ti. | 1052 |
| #6 | exp Poverty/ or Poverty Areas/ or ((economic* or social* or financial*) adj1 (disadvantage* or vulnerabl* or low or poor)).ab,ti. or (farmer* or rural or village* or construction worker* or sanitation worker* or unemployed or poor or poverty or underserv* or low-income or low income).ab,ti. | 11513 |
| #7 | exp Disabled/ or exp Vision Disorders/ or exp Hearing Disorders/ or ((Mobility or physical or vision or visual or hearing) adj1 (disab* or impair* or limit* or loss)).ab,ti. or (wheelchair or handicap* or disabled or deaf*).ab,ti. | 3223 |
| #8 | (Factor* or influenc* or associat* or correlat* or facilitator* or motivat* or enabl* or promot* or facilitat* or predict* or determin* or barrier* or preclud* or challeng*).ab,ti. | 113848 |
| #9 | #3 or #4 or #5 or #6 or #7 | 17818 |
| #10 | 1 and 2 and 8 and 9 | 22 |
| APA PsycInfo on May 4, 2023 | | |
| #1 | exp Hepatitis/ or (hepatitis B or HBV or hep B or CHB).ab,ti. | 3851 |
| #2 | exp Screening/ or exp Testing/ or exp Vaccination Attitudes/ or exp Vaccination/ or exp "Continuum of Care"/ or exp Health Care Access/ or exp Health Care Utilization/ or (screen* or test* or vaccin* or immun* or linkage to care or linkage-to-care or linkage or linking or care continuum or care or refer* or follow up or follow-up or monitor* or surveillance or examination* or assessment* or visit* or attend* or clinic* or hospital* or hepatolog* or physician* or specialist* or provider* or treatment* or therap* or manag* or counseling or counselling or service* or healthcare or health care).ab,ti. | 3132161 |
| #3 | exp Marginalization/ or exp Marginalized Groups/ or exp Health Disparities/ or exp Disadvantaged/ or ((marginalized or marginalised or hard to reach or seldom heard or vulnerabl* or exclude* or segregate* or discriminat* or disadvantage* or minority) adj1 (people or patient* or population* or group* or sample* or participant*)).ab,ti. | 52928 |
| #4 | exp Homeless Mentally Ill/ or exp Homeless/ or exp Homeless Youth/ or (homeless* or home-less or shelter* or unhoused or unstab* hous* or street* or vagabond* or hobo* or drifter*).ab,ti. | 30945 |
| #5 | exp Migrant Workers/ or exp Migrant Farm Workers/ or (Migrant* adj1 (worker* or farmworker*)).ab,ti. or (migrant domestic worker* or migrant farmworker* or migrant construction worker*).ab,ti. | 1439 |
| #6 | exp Lower Income Level/ or exp Poverty Areas/ or exp Poverty/ or ((economic* or social* or financial*) adj1 (disadvantage* or vulnerabl* or low or poor)).ab,ti. or (farmer* or rural or village* or construction worker* or sanitation worker* or unemployed or poor or poverty or underserv* or low-income or low income).ab,ti. | 231849 |
| #7 | exp Disabled Personnel/ or exp Vision Disorders/ or exp Hearing Disorders/ or ((Mobility or physical or vision or visual or hearing) adj1 (disab* or impair* or limit* or loss)).ab,ti. or (wheelchair or handicap* or disabled or deaf*).ab,ti. | 103152 |
| #8 | (Factor* or influenc* or associat* or correlat* or facilitator* or motivat* or enabl* or promot* or facilitat* or predict* or determin* or barrier* or preclud* or challeng*).ab,ti. | 2964189 |
| #9 | #3 or #4 or #5 or #6 or #7 | 396024 |
| #10 | #1 AND #2 AND #8 AND #9 | 447 |
| CINAHL Ultimate on May 4, 2023 | | |
| #1 | (MH "Hepatitis B, Chronic") OR (MH "Hepatitis B+") | 10,861 |
| #2 | TI ( hepatitis B or HBV or hep B or CHB ) OR AB ( hepatitis B or HBV or hep B or CHB ) | 14,899 |
| #3 | (MH "Vaccination Hesitancy") OR (MH "Vaccination Refusal+") OR (MH "Vaccination Status") OR (MH "Immunization-Vaccination Administration (Iowa NIC)") OR (MH "Vaccination Coverage") OR (MH "Immunization+") | 33,582 |
| #4 | (MH "Point-of-Care Testing+") | 4,584 |
| #5 | (MH "Continuity of Patient Care+") | 24,802 |
| #6 | (MH "Patient Care+") | 903,275 |
| #7 | TI ( screen* or test* or vaccin* or immun* or linkage to care or linkage-to-care or linkage or linking or care continuum or care or refer* or follow up or follow-up or monitor* or surveillance or examination* or assessment* or visit* or attend* or clinic* or hospital* or hepatolog* or physician* or specialist* or provider* or treatment* or therap* or manag* or counseling or counselling or service* or healthcare or health care ) OR AB ( screen* or test* or vaccin* or immun* or linkage to care or linkage-to-care or linkage or linking or care continuum or care or refer* or follow up or follow-up or monitor* or surveillance or examination* or assessment* or visit* or attend* or clinic* or hospital* or hepatolog* or physician* or specialist* or provider* or treatment* or therap* or manag* or counseling or counselling or service* or healthcare or health care ) | 4,400,886 |
| #8 | (MH "Healthcare Disparities") OR (MH "Health Inequities") | 19,489 |
| #9 | TI ( marginalized or marginalised or hard to reach or seldom heard or vulnerabl* or exclude* or segregate* or discriminat* or disadvantage* or minority ) OR AB ( marginalized or marginalised or hard to reach or seldom heard or vulnerabl* or exclude* or segregate* or discriminat* or disadvantage* or minority ) | 224,620 |
| #10 | (MH "Homeless Persons") OR (MH "Homelessness") | 10,507 |
| #11 | TI ( homeless* or home-less or shelter* or unhoused or unstab* hous* or street* or vagabond* or hobo* or drifter* ) OR AB ( homeless* or home-less or shelter* or unhoused or unstab* hous* or street* or vagabond* or hobo* or drifter* ) | 22,380 |
| #12 | (MH "Farmworkers") | 3,824 |
| #13 | (MH "Transients and Migrants") | 5,956 |
| #14 | TI ( migrant domestic worker* or migrant farmworker* or migrant construction worker* ) OR AB ( migrant domestic worker* or migrant farmworker* or migrant construction worker* ) | 412 |
| #15 | (MH "Poverty+") OR (MH "Poverty Areas") OR (MH "Social Deprivation") | 31,189 |
| #16 | TI ( farmer* or rural or village* or construction worker* or sanitation worker* or unemployed or poor or poverty or underserv* or low-income or low income ) OR AB ( farmer* or rural or village* or construction worker* or sanitation worker* or unemployed or poor or poverty or underserv* or low-income or low income ) | 301,833 |
| #17 | TI ( economic* or social* or financial* ) AND TI ( disadvantage* or vulnerabl* or low or poor ) | 2,850 |
| #18 | AB ( economic* or social* or financial* ) AND AB ( disadvantage* or vulnerabl* or low or poor ) | 77,941 |
| #19 | (MH "Parents with Disabilities") | 590 |
| #20 | (MH "Vision Disorders+") OR (MH "Deaf-Blind Disorders+") | 21,278 |
| #21 | (MH "Hearing Loss, Functional") | 86 |
| #22 | TI ( Mobility or physical or vision or visual or hearing ) AND TI ( Mobility or physical or vision or visual or hearing ) | 141,985 |
| #23 | AB ( disab* or impair* or limit* or loss ) AND AB ( disab* or impair* or limit* or loss ) | 757,402 |
| #24 | TI ( wheelchair or handicap* or disabled or deaf* ) OR AB ( wheelchair or handicap* or disabled or deaf* ) | 37,763 |
| #25 | TI ( Factor* or influenc* or associat* or correlat* or facilitator* or motivat* or enabl* or promot* or facilitat* or predict* or determin* or barrier* or preclud* or challeng* ) OR AB ( Factor* or influenc* or associat* or correlat* or facilitator* or motivat* or enabl* or promot* or facilitat* or predict* or determin* or barrier* or preclud* or challeng* ) | 2,935,064 |
| #26 | #1 or #2 | 17,112 |
| #27 | #3 or #4 or #5 or #6 or #7 | 4,674,811 |
| #28 | #8 or #9 | 240,052 |
| #29 | #10 or #11 | 24,811 |
| #30 | #12 or #13 or #14 | 9,354 |
| #31 | #15 or #16 or #17 or #18 | 356,593 |
| #32 | #19 or #20 or #21 or #22 or #23 or #24 | 894,706 |
| #33 | #28 or #29 or #30 or #32 or #32 | 1,343,759 |
| #35 | #25 AND #26 AND #27 AND #33 | 2,100 |
| ProQuest Health & Medicine Collection May 4, 2023 | | |
| #1 | subject(hepatitis B or hepatitis B virus) OR title(hepatitis B or HBV or hep B or CHB) OR abstract(hepatitis B or HBV or hep B or CHB) | 41,222 |
| #2 | subject(screening or mass screening or Point-of-Care Testing or vaccination or vaccination refusal or vaccination hesitancy or vaccination coverage or vaccines or immunization or continuity of patient care or patient care or health care or medical care) OR title(screen* or test* or vaccin* or immun* or linkage to care or linkage-to-care or linkage or linking or care continuum or care or refer* or follow up or follow-up or monitor* or surveillance or examination* or assessment* or visit* or attend* or clinic* or hospital* or hepatolog* or physician* or specialist* or provider* or treatment* or therap* or manag* or counseling or counselling or service* or healthcare or health care) OR abstract(screen* or test* or vaccin* or immun* or linkage to care or linkage-to-care or linkage or linking or care continuum or care or refer* or follow up or follow-up or monitor* or surveillance or examination* or assessment* or visit* or attend* or clinic* or hospital* or hepatolog* or physician* or specialist* or provider* or treatment* or therap* or manag* or counseling or counselling or service* or healthcare or health care) | 8,190,273 |
| #3 | subject(Vulnerable Populations or health disparity or marginalized or marginalization or special populations) OR title(marginalized or marginalised or hard to reach or seldom heard or vulnerabl* or exclude* or segregate* or discriminat* or disadvantage* or minority) OR abstract(marginalized or marginalised or hard to reach or seldom heard or vulnerabl* or exclude* or segregate* or discriminat* or disadvantage* or minority) | 460,925 |
| #4 | subject(homeless person or homelessness or Homeless Shelters) OR title(homeless* or home-less or shelter* or unhoused or unstab* hous* or street* or vagabond* or hobo* or drifter*) OR abstract(homeless* or home-less or shelter* or unhoused or unstab* hous* or street* or vagabond* or hobo* or drifter*) | 85,119 |
| #5 | subject(migrant worker or Migrant Farm Workers) OR title(migrant domestic worker* or migrant farmworker* or migrant construction worker*) OR abstract(migrant domestic worker* or migrant farmworker* or migrant construction worker*) | 5,207 |
| #6 | title(Migrant*) AND title(worker* or farmworker*) | 1,598 |
| #7 | abstract(Migrant*) AND abstract(worker* OR farmworker*) | 3,193 |
| #8 | subject(Low Socioeconomic Status or Poverty or Poverty Areas or extreme poverty) OR title(farmer* or rural or village* or construction worker* or sanitation worker* or unemployed or poor or poverty or underserv* or low-income or low income) OR abstract(farmer* or rural or village* or construction worker* or sanitation worker* or unemployed or poor or poverty or underserv* or low-income or low income) | 683,097 |
| #9 | title(economic* or social* or financial*) AND title(disadvantage* or vulnerabl* or low or poor) | 4,315 |
| #10 | abstract(economic* OR social* OR financial*) AND abstract(disadvantage* OR vulnerabl* OR low OR poor) | 179,412 |
| #11 | subject(Disabled Persons or Vision Disorders or Hearing Loss or Hearing impairment or Hearing Disorders) OR title(wheelchair or handicap* or disabled or deaf*) OR abstract(wheelchair or handicap* or disabled or deaf*) | 105,519 |
| #12 | title(Mobility or physical or vision or visual or hearing) AND title(disab* or impair* or limit* or loss) | 23,073 |
| #13 | abstract(Mobility OR physical OR vision OR visual OR hearing) AND abstract(disab* OR impair* OR limit* OR loss) | 173,159 |
| #14 | title(Factor* or influenc* or associat* or correlat* or facilitator* or motivat* or enabl* or promot* or facilitat* or predict* or determin* or barrier* or preclud* or challeng*) OR abstract(Factor* or influenc* or associat* or correlat* or facilitator* or motivat* or enabl* or promot* or facilitat* or predict* or determin* or barrier* or preclud* or challeng*) | 5,960,924 |
| #15 | #3 or #4 or #5 or #6 or #7 or #8 or #9 or #10 or #11 or #12 or #13 or #14 | 1,460,695 |
| #16 | #1 AND #2 AND #14 AND #15 | 3,373 |
| #17 | NOT Dissertations & Theses NOT (Case Study AND Commentary AND Correspondence AND Editorial AND News AND Letter To The Editor AND Conference Proceeding)  Limit to Health & Medical Collection | 2934 |
| British Nursing Index on May 4, 2023 | | |
| #1 | subject(hepatitis B or hepatitis B virus) OR title(hepatitis B or HBV or hep B or CHB) OR abstract(hepatitis B or HBV or hep B or CHB) | 2,170 |
| #2 | subject(screening or mass screening or Point-of-Care Testing or vaccination or vaccination refusal or vaccination hesitancy or vaccination coverage or vaccines or immunization or continuity of patient care or patient care or health care or medical care) OR title(screen* or test* or vaccin* or immun* or linkage to care or linkage-to-care or linkage or linking or care continuum or care or refer* or follow up or follow-up or monitor* or surveillance or examination* or assessment* or visit* or attend* or clinic* or hospital* or hepatolog* or physician* or specialist* or provider* or treatment* or therap* or manag* or counseling or counselling or service* or healthcare or health care) OR abstract(screen* or test* or vaccin* or immun* or linkage to care or linkage-to-care or linkage or linking or care continuum or care or refer* or follow up or follow-up or monitor* or surveillance or examination* or assessment* or visit* or attend* or clinic* or hospital* or hepatolog* or physician* or specialist* or provider* or treatment* or therap* or manag* or counseling or counselling or service* or healthcare or health care) | 724,615 |
| #3 | subject(Vulnerable Populations or health disparity or marginalized or marginalization or special populations) OR title(marginalized or marginalised or hard to reach or seldom heard or vulnerabl* or exclude* or segregate* or discriminat* or disadvantage* or minority) OR abstract(marginalized or marginalised or hard to reach or seldom heard or vulnerabl* or exclude* or segregate* or discriminat* or disadvantage* or minority) | 36,556 |
| #4 | subject(homeless person or homelessness or Homeless Shelters) OR title(homeless* or home-less or shelter* or unhoused or unstab* hous* or street* or vagabond* or hobo* or drifter*) OR abstract(homeless* or home-less or shelter* or unhoused or unstab* hous* or street* or vagabond* or hobo* or drifter*) | 5,184 |
| #5 | subject(migrant worker or Migrant Farm Workers) OR title(migrant domestic worker* or migrant farmworker* or migrant construction worker*) OR abstract(migrant domestic worker* or migrant farmworker* or migrant construction worker*) | 339 |
| #6 | title(Migrant*) AND title(worker* or farmworker*) | 148 |
| #7 | abstract(Migrant*) AND abstract(worker* OR farmworker*) | 335 |
| #8 | subject(Low Socioeconomic Status or Poverty or Poverty Areas or extreme poverty) OR title(farmer* or rural or village* or construction worker* or sanitation worker* or unemployed or poor or poverty or underserv* or low-income or low income) OR abstract(farmer* or rural or village* or construction worker* or sanitation worker* or unemployed or poor or poverty or underserv* or low-income or low income) | 46,602 |
| #9 | title(economic* or social* or financial*) AND title(disadvantage* or vulnerabl* or low or poor) | 323 |
| #10 | abstract(economic* OR social* OR financial*) AND abstract(disadvantage* OR vulnerabl* OR low OR poor) | 14,311 |
| #11 | subject(Disabled Persons or Vision Disorders or Hearing Loss or Hearing impairment or Hearing Disorders) OR title(wheelchair or handicap* or disabled or deaf*) OR abstract(wheelchair or handicap* or disabled or deaf*) | 8,796 |
| #12 | title(Mobility or physical or vision or visual or hearing) AND title(disab* or impair* or limit* or loss) | 1,113 |
| #13 | abstract(Mobility OR physical OR vision OR visual OR hearing) AND abstract(disab* OR impair* OR limit* OR loss) | 10,765 |
| #14 | title(Factor* or influenc* or associat* or correlat* or facilitator* or motivat* or enabl* or promot* or facilitat* or predict* or determin* or barrier* or preclud* or challeng*) OR abstract(Factor* or influenc* or associat* or correlat* or facilitator* or motivat* or enabl* or promot* or facilitat* or predict* or determin* or barrier* or preclud* or challeng*) | 318,766 |
| #15 | #3 or #4 or #5 or #6 or #7 or #8 or #9 or #10 or #11 or #12 or #13 or #14 | 101,139 |
| #16 | #1 AND #2 AND #14 AND #15 | 151 |
| Scopus on May 4, 2023 | | |
| #1 | TITLE-ABS-KEY ( hepatitis AND b OR hbv OR hep AND b OR chb ) | 187,865 |
| #2 | TITLE-ABS-KEY ( screen* OR test* OR vaccin* OR immun* OR linkage AND to AND care OR linkage-to-care OR linkage OR linking OR care AND continuum OR care OR refer* OR follow AND up OR follow-up OR monitor* OR surveillance OR examination* OR assessment* OR visit* OR attend* OR clinic* OR hospital* OR hepatolog* OR physician* OR specialist* OR provider* OR treatment* OR therap* OR manag* OR counseling OR counselling OR service* OR healthcare OR health AND care ) | 993,782 |
| #3 | ( TITLE-ABS-KEY ( marginalized OR marginalised OR hard AND to AND reach OR seldom AND heard OR vulnerabl* OR exclude* OR segregate* OR discriminat* OR disadvantage* OR minority ) AND TITLE-ABS-KEY ( people OR patient* OR population* OR group* OR sample* OR participant* ) ) | 1,163 |
| #4 | TITLE-ABS-KEY ( homeless* OR home-less OR shelter* OR unhoused OR unstab* AND hous* OR street* ORvagabond* OR hobo* OR drifter* ) | 21,011 |
| #5 | ( TITLE-ABS-KEY ( migrant* ) AND TITLE-ABS-KEY ( worker* OR farmworker* ) ) | 19,225 |
| #6 | TITLE-ABS-KEY ( migrant AND domestic AND worker* OR migrant AND farmworker* OR migrant AND construction AND worker* ) | 109 |
| #7 | ( TITLE-ABS-KEY ( economic* OR social* OR financial* ) AND TITLE-ABS-KEY ( disadvantage* OR vulnerabl* OR low OR poor ) ) | 782,536 |
| #8 | TITLE-ABS-KEY ( farmer* OR rural OR village* OR construction AND worker* OR sanitation AND worker* OR unemployed OR poor OR poverty OR underserv* OR low-income OR low AND income ) | 6,285 |
| #9 | ( TITLE-ABS-KEY ( mobility OR physical OR vision OR visual OR hearing ) AND TITLE-ABS-KEY ( disab* OR impair* OR limit* OR loss ) ) | 1,256,421 |
| #10 | TITLE-ABS-KEY ( wheelchair OR handicap* OR disabled OR deaf* ) | 279,206 |
| #11 | ( TITLE-ABS-KEY ( mobility OR physical OR vision OR visual OR hearing ) AND TITLE-ABS-KEY ( disab* OR impair* OR limit* OR loss ) ) | 1,256,421 |
| #12 | TITLE-ABS-KEY ( wheelchair OR handicap* OR disabled OR deaf* ) | 279,206 |
| #13 | TITLE-ABS-KEY ( factor* OR influenc* OR associat* OR correlat* OR facilitator* OR motivat* OR enabl* OR promot* OR facilitat* OR predict* OR determin* OR barrier* OR preclud* OR challeng* ) | 37,730,928 |
| #14 | #3 or #4 or #5 or #6 or #7 or #8 or #9 or #10 or #11 or #12 or #13 | 2,212,549 |
| #15 | #1 AND #2 AND #13 AND #14 | 837 |
| Cochrane library on May 4, 2023 | | |
| #1 | MeSH descriptor: [Hepatitis B] explode all trees | 3,553 |
| #2 | MeSH descriptor: [Hepatitis B virus] explode all trees | 1,115 |
| #3 | (hepatitis B or HBV or hep B or CHB):ti,ab,kw | 12,678 |
| #4 | #1 or #2 or #3 | 12,678 |
| #5 | MeSH descriptor: [Mass Screening] explode all trees | 5,346 |
| #6 | MeSH descriptor: [Vaccination Refusal] explode all trees | 23 |
| #7 | MeSH descriptor: [Vaccination Hesitancy] explode all trees | 12 |
| #8 | MeSH descriptor: [Vaccination Coverage] explode all trees | 60 |
| #9 | MeSH descriptor: [Continuity of Patient Care] explode all trees | 35,764 |
| #10 | MeSH descriptor: [Point-of-Care Testing] explode all trees | 155 |
| #11 | MeSH descriptor: [Patient Care] explode all trees | 92,324 |
| #12 | (screen* or test* or vaccin* or immun* or linkage to care or linkage-to-care or linkage or linking or care continuum or care or refer* or follow up or follow-up or monitor* or surveillance or examination* or assessment* or visit* or attend* or clinic* or hospital* or hepatolog* or physician* or specialist* or provider* or treatment* or therap* or manag* or counseling or counselling or service* or healthcare or health care):ti,ab,kw | 171,3884 |
| #13 | #5 or #6 or #7 or #8 or #9 or #10 or #11 or #12 | 171,6502 |
| #14 | MeSH descriptor: [Vulnerable Populations] explode all trees | 456 |
| #15 | MeSH descriptor: [Health Disparate, Minority and Vulnerable Populations] explode all trees | 7,051 |
| #16 | (marginalized or marginalised or hard to reach or seldom heard or vulnerabl* or exclude* or segregate* or discriminat* or disadvantage* or minority):ti,ab,kw AND (people or patient* or population* or group* or sample* or participant*):ti,ab,kw | 62,602 |
| #17 | MeSH descriptor: [Ill-Housed Persons] explode all trees | 480 |
| #18 | (homeless* or home-less or shelter* or unhoused or unstab* hous* or street* or vagabond* or hobo* or drifter*):ti,ab,kw | 2,322 |
| #19 | MeSH descriptor: [Transients and Migrants] explode all trees | 105 |
| #20 | (Migrant*):ti,ab,kw AND (worker* or farmworker*):ti,ab,kw | 96 |
| #21 | (migrant domestic worker* or migrant farmworker* or migrant construction worker*):ti,ab,kw | 17 |
| #22 | MeSH descriptor: [Poverty] explode all trees | 2,258 |
| #23 | (disadvantage* or vulnerabl* or low or poor):ti,ab,kw AND (economic* or social* or financial*):ti,ab,kw | 22,886 |
| #24 | (farmer* or rural or village* or construction worker* or sanitation worker* or unemployed or poor or poverty or underserv* or low-income or low income):ti,ab,kw | 72,922 |
| #25 | MeSH descriptor: [Disabled Persons] explode all trees | 1,510 |
| #26 | MeSH descriptor: [Vision Disorders] explode all trees | 2,199 |
| #27 | MeSH descriptor: [Hearing Loss] explode all trees | 1,609 |
| #28 | (Mobility or physical or vision or visual or hearing):ti,ab,kw AND (disab* or impair* or limit* or loss):ti,ab,kw | 77,289 |
| #29 | (wheelchair or handicap* or disabled or deaf*):ti,ab,kw | 8,152 |
| #30 | (Factor* or influenc* or associat* or correlat* or facilitator* or motivat* or enabl* or promot* or facilitat* or predict* or determin* or barrier* or preclud* or challeng*):ti,ab,kw | 928,551 |
| #31 | #14 or #15 or #16 or #17 or #18 or #19 or #20 or #21 or #22 or #23 or #24 or #25 or #26 or #27 or #28 or #29 | 218,630 |
| #32 | #4 AND #13 AND #30 AND #31 | 875 |
| 中国知网on May 4, 2023 | | |
|  | SU=(乙肝 + 乙型肝炎 + HBV) and SU=(筛查 + 疫苗防疫 + 预防 + 防控 + 检查 + 监测 + 治疗 + 随访 + 医疗 + 医院 + 诊所 + 护理) and SU=(边缘 + 健康差异 + 流浪 + 乞讨 + 农民工 + 农民 + 农村 + 建筑工 + 清洁工 + 家庭佣工 + 低收入 + 贫困 + 残疾 + 残障) | 294 |
