# Supplementary material for: Disparities in hepatitis B virus healthcare service access among marginalised poor populations: a mixed-method systematic review
Source: Infect Dis Poverty. 2024 Aug 9;13:58. doi: 10.1186/s40249-024-01225-0 (PMC11312201; doi:10.1186/s40249-024-01225-0)
Supplement: Supplementary file 2 — Supplementary Material 2. [file 40249_2024_1225_MOESM2_ESM.docx]

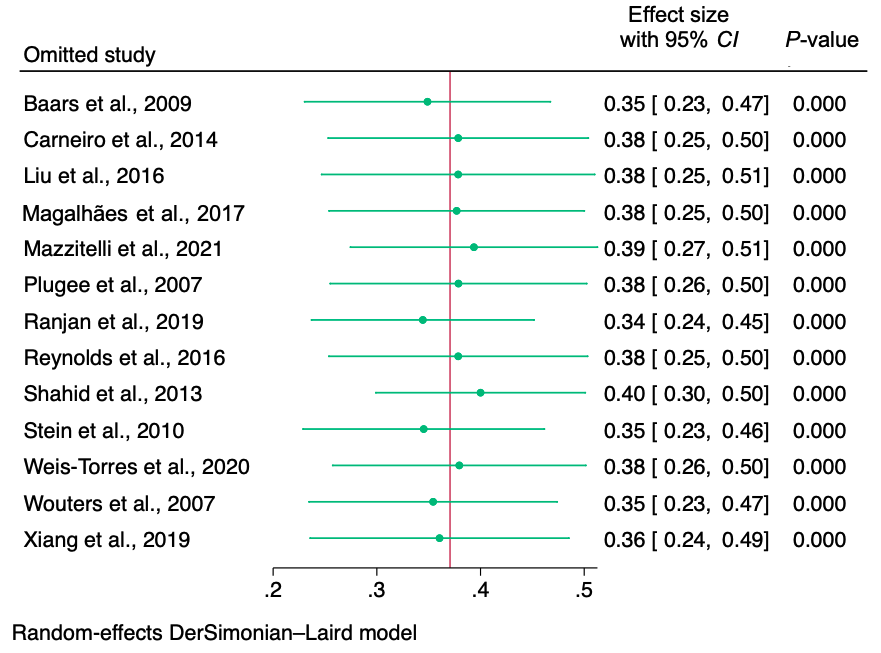


**Fig.** Sensitivity analysis of pooled rate of HBV vaccination among marginalized poor populations.

**Table 1** Appraisal of the methodological quality of included quantitative descriptive studies (*n* = 17)

| **Study** | **Clear research questions presented** | **The collected data address research questions** | **Sampling strategy can address research questions** | **Sample is representative to target population** | **Measurements are appropriate** | **Risk of nonresponse bias is low** | **Statistical analyses are appropriate** | **Score**  **(Range 1 to 7)** | **% Score** |
| --- | --- | --- | --- | --- | --- | --- | --- | --- | --- |
| Baars et al., 2009 [23] | Yes | Yes | Yes | Yes | No | Yes | Yes | 6 | 86% |
| Carneiro et al., 2014 [24] | Yes | Yes | Yes | Yes | Yes | Yes | Yes | 7 | 100% |
| Jung et al., 2010 [26] | Yes | Yes | Yes | Yes | Yes | No | Yes | 6 | 86% |
| Liu et al., 2016 [27] | Yes | Yes | Yes | Yes | Yes | Yes | Yes | 7 | 100% |
| Magalhães et a., 2017 [28] | Yes | Yes | Yes | Yes | No | Can’t tell | Yes | 5 | 71% |
| Mayanja et al., 2019 [29] | Yes | Yes | Yes | Yes | Yes | Yes | Yes | 7 | 100% |
| Mazzitelli et al., 2021 [30] | Yes | Yes | Can’t tell | Yes | Yes | Yes | No | 5 | 71% |
| Nyamathi et al., 2009 [31] | Yes | Yes | Yes | Yes | Can’t tell | Yes | Yes | 6 | 86% |
| Nyamathi et al., 2015 [32] | Yes | Yes | Yes | Yes | Yes | Yes | Yes | 7 | 100% |
| Plugee et al., 2007 [33] | Yes | Yes | Yes | Yes | No | Yes | Yes | 6 | 86% |
| Ranjan et al., 2019 [34] | Yes | Yes | Yes | Yes | No | Can’t tell | Yes | 5 | 71% |
| Reynolds et al., 2016 [35] | Yes | Yes | Can’t tell | Can’t tell | Yes | Can’t tell | Yes | 4 | 57% |
| Sahajian et al., 2010 [16] | Yes | Yes | Yes | Yes | Yes | Yes | Yes | 7 | 100% |
| Shahid et al., 2013 [37] | Yes | Yes | No | Yes | Can’t tell | Can’t tell | No | 3 | 43% |
| Stein et al., 2010 [38] | Yes | Yes | Yes | Yes | Yes | Can’t tell | Yes | 6 | 86% |
| Weis-Torres et al., 2020 [39] | Yes | Yes | No | Yes | Yes | Yes | Yes | 6 | 86% |
| Wong et al., 2018 [40] | Yes | Yes | Yes | Yes | Yes | Can’t tell | No | 5 | 71% |
| Wouters et al., 2007 [41] | Yes | Yes | No | Yes | Yes | Can’t tell | Yes | 5 | 71% |
| Xiang et al., 2019 [42] | Yes | Yes | Yes | Yes | No | Yes | Yes | 6 | 86% |

**Table 2** Appraisal of the methodological quality of included qualitative studies (*n* = 2)

| **Study** | **Clear research questions presented** | **The collected data address research questions** | **Qualitative approach is appropriate to address research question** | **Qualitative methods are adequate** | **Findings are adequately derived from data** | **Interpretation of results are sufficiently substantiated by data** | **There is coherence between qualitative data sources, collection, analysis and interpretation** | **Score**  **(Range 1 to 7)** | **% Score** |
| --- | --- | --- | --- | --- | --- | --- | --- | --- | --- |
| Freeland et al., 2021 [25] | Yes | Yes | Yes | Yes | Yes | Yes | Yes | 7 | 100% |
| Stantilli et al., 2018 [36] | Yes | Yes | Yes | Yes | No | No | Can’t tell | 4 | 57% |
